# Water-participated mild oxidation of ethane to acetaldehyde

**DOI:** 10.1038/s41467-024-46884-7

**Published:** 2024-03-22

**Authors:** Bin Li, Jiali Mu, Guifa Long, Xiangen Song, Ende Huang, Siyue Liu, Yao Wei, Fanfei Sun, Siquan Feng, Qiao Yuan, Yutong Cai, Jian Song, Wenrui Dong, Weiqing Zhang, Xueming Yang, Li Yan, Yunjie Ding

**Affiliations:** 1 Dalian National Laboratory for Clean Energy, Dalian Institute of Chemical Physics, Chinese Academy of Sciences, Dalian, China; 2 University of Chinese Academy of Sciences, Beijing, China; 3 Guangxi Key Laboratory of Chemistry and Engineering of Forest Products, School of Chemistry and Chemical Engineering, Guangxi Minzu University, Nanning, China; 4 Shanghai Synchrotron Radiation Facility, Shanghai Advanced Research Institute, Chinese Academy of Sciences, Shanghai, China; 5 State Key Laboratory of Molecular Reaction Dynamics, Dalian Institute of Chemical Physics, Chinese Academy of Sciences, Dalian, China; 6 Hefei National Laboratory, Hefei, China; 7 Department of Chemistry, Southern University of Science and Technology, Shenzhen, China; 8 State Key Laboratory of Catalysis, Dalian Institute of Chemical Physics, Chinese Academy of Sciences, Dalian, China

**Keywords:** Heterogeneous catalysis, Catalytic mechanisms, Catalyst synthesis

## Abstract

The direct conversion of low alkane such as ethane into high-value-added chemicals has remained a great challenge since the development of natural gas utilization. Herein, we achieve an efficient one-step conversion of ethane to C_2_ oxygenates on a Rh_1_/AC-SNI catalyst under a mild condition, which delivers a turnover frequency as high as 158.5 h^−1^. ^18^O isotope-GC–MS shows that the formation of ethanol and acetaldehyde follows two distinct pathways, where oxygen and water directly participate in the formation of ethanol and acetaldehyde, respectively. In situ formed intermediate species of oxygen radicals, hydroxyl radicals, vinyl groups, and ethyl groups are captured by laser desorption ionization/time of flight mass spectrometer. Density functional theory calculation shows that the activation barrier of the rate-determining step for acetaldehyde formation is much lower than that of ethanol, leading to the higher selectivity of acetaldehyde in all the products.

## Abstract (web summary)

The direct conversion of low alkane-like ethane into high-value chemicals has posed a significant challenge. Herein, the authors successfully accomplish a one-step conversion of ethane to C2 oxygenates using a Rh single-atom catalyst under mild conditions.

## Introduction

With the growing demand for energy and chemical products in contemporary society, the utilization of natural gas has been attracting more and more attention. At present, most studies on the direct conversion of low-carbon alkanes at relatively lower temperatures are focused on methane^1–14^. Ethane (C_2_H_6_) is the second major component in shale gas, which features a high C–H bond energy of 423.29 kJ mol^−1^ and thus high-temperature range of 700–1000 K is usually needed for its cracking and dehydrogenation into ethylene^15–20^. However, the high-temperature process often brings about significant side reactions, such as coke or deactivation of the catalyst, and huge energy consumption. Therefore, it is of great significance to develop a direct conversion path of ethane under lower temperature conditions. There have been not so many reports on the direct conversion of ethane under mild conditions^2–4,21–24^. Hutchings et al. found that ethane could be directly converted to ethanol and ethyl hydroperoxide, which would be further oxidized to acetaldehyde and acetic acid at 323 K on Fe-ZSM-5 catalyst using H_2_O_2_^21^. Martin et al. realized low-temperature oxidation of ethane to oxygenates by oxygen over Ir-cluster catalysts in the presence of CO^24^. Furthermore, the indispensable role of CO in the reaction system has also been studied^25,26^. Nevertheless, there are still two major challenges in the current field of direct oxidation of alkanes: (a) to study whether the active sites provided by single-atom catalysts are different from the pathways used by traditional catalysts to activate alkanes and (b) to figure out the cooperative oxidation mechanism of H_2_O and O_2_ in alkane activation.

Heterogeneous single-metal-site catalysts (HSMSCs) have recently emerged as an important class of high-efficiency catalysts with almost 100% atomic utilization and unique properties^27,28^, which can ideally bridge the gap between heterogeneous and homogeneous catalysis and offer a platform for understanding the nature of active sites at the molecular level^29^. The relationships between coordination atoms and metal centers determine the electronic state and geometry of a single-metal site and further the performance of catalytic reactions. In recent years, the gradual development of tuning the local coordination environment of single-metal-sites by doping N atoms on carbon carriers has emerged^30,31^. Additionally, sulfur as an electron-donating ligand can also form a coordination bond with the active center, influencing electron cloud density and thus enhancing reaction activity^32,33^. We previously revealed that S species can promote the activity of methanol carbonylation by coordination with single-Rh-site^34,35^. Furthermore, the crucial role of I species in achieving atomic dispersion of Rh nanoparticles (NPs) was investigated^27,36^. Introducing multifarious coordination atoms (S, N, and I) onto the active carbon support as anchoring sites to immobile single-metal-site offers a prospective pathway for synthesizing HSMSCs.

Herein, we first show that the single-Rh-site bound on S, N, and I-doped activated carbon (Rh_1_/AC-SNI) in the form of Rh mononuclear complex efficiently catalyzes the direct conversion of ethane to C_2_ oxygenate products using an O_2_ oxidizing agent at 423 K in aqueous solution. Moreover, the turnover frequency (TOF) of the oxygenate products can reach as high as 158.5 h^−1^ on Rh_1_/AC-SNI, which is the highest activity compared with the reported results in the literature so far. The ethane activation on the Rh_1_ was systematically investigated by a variety of characterizations, which revealed that O_2_ and H_2_O could directly participate in the reaction of ethane oxidation. Isotope-GC–MS results show that the formation of ethanol and acetaldehyde follows two different reaction pathways, and the O source of ethanol product originates from O_2_, while H_2_O directly involved in the generation process of acetaldehyde. Experimental results combined with density functional theoretical (DFT) calculations show that the energy barrier for the production of ethanol at the Rh active site is higher than the energy barrier for the production of acetaldehyde.

## Results

### Catalyst characterization

**Fig. 1:**
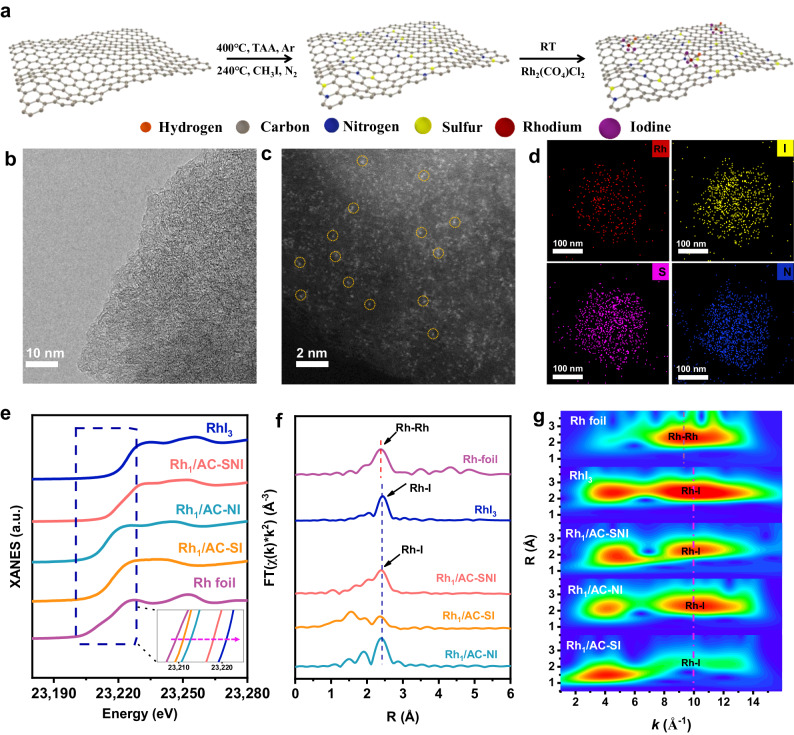
Synthesis and structural characterization of samples. **a** Schematic illustration of the preparation of Rh_1_/AC-NI, Rh_1_/AC-SI, and Rh_1_/AC-SNI catalysts. **b** HR**-**TEM images of fresh Rh_1_/AC-SNI. **c** HAADF-STEM images of fresh Rh_1_/AC-SNI. **d** HAADF-EDS-mapping of fresh Rh_1_/AC-SNI. **e** XANES spectra for Rh foil, RhI_3_, Rh_1_/AC-NI, Rh_1_/AC-SI, and Rh_1_/AC-SNI. **f** The experimental curve of *k*^2^-weight EXAFS spectra in R-space of Rh foil, RhI_3_, Rh_1_/AC-NI, Rh_1_/AC-SI, and Rh_1_/AC-SNI. **g** The wavelet transforms contour plots of *k*^2^-weighted χ(k) EXAFS signals of Rh foil, RhI_3_, Rh_1_/AC-NI, Rh_1_/AC-SI, and Rh_1_/AC-SNI.

The Rh_1_/AC-SNI catalyst was synthesized based on the strong coordination interaction between a single Rh ion and N, S, and I ligands on the AC (see details in the Experimental section). As shown in Fig. 1a, activated carbon (AC) was first co-doped with S and N, followed by I incorporation. Subsequently, the modified AC was coordinated with Rh at ambient conditions to get the final product. For comparison, the samples with single N or S doping were prepared using the same protocol, which is denoted as Rh_1_/AC-SI and Rh_1_/AC-NI, respectively. We explored the excellent microstructural properties of Rh_1_/AC-SNI and comparative catalysts by scanning electron microscopy (SEM), nitrogen adsorption-desorption, thermogravimetric, and Raman spectroscopy experiments (Supplementary Figs. 1–4 and Supplementary Table 1)^37,38^. In line with the X-ray diffraction (XRD) results (Supplementary Fig. 5), no clusters or NPs were observed by the high-resolution transmission electron microscopy (HR-TEM) (Fig. 1b and Supplementary Fig. 6). The aberration-corrected high-angle annular dark-field scanning transmission electron microscopy (AC HAADF-STEM) image further indicates the atomic dispersion of Rh ions in the carbon matrix according to the prominent Z-contrast difference between Rh and C atoms and the absence of Rh NPs or clusters (Fig. 1c and Supplementary Fig. 7). Energy dispersive spectroscopy (EDS) mapping confirms the uniform distribution of S, N, I, and Rh on Rh_1_/AC-SNI (Fig. 1d). Similar structural and morphological information was validated on the Rh_1_/AC-SI and Rh_1_/AC-NI catalysts (Supplementary Figs. 1–7).

The X-ray absorption near-edge structure (XANES) spectra exhibit that all the absorption of Rh on Rh_1_/AC-NI, Rh_1_/AC-SI, and Rh_1_/AC-SNI is located between those of RhI_3_ and Rh foil, revealing that the valence state of Rh^δ+^ species is between 0 and +3 (Fig. 1e). Based on Fig. 1f, g, Rh–Rh (8.9 Å^−1^) and Rh-I (9.6 Å^−^^1^) can be distinguished, further verifying the absence of Rh NPs. Subsequently, the detailed extended X-ray absorption fine structure (EXAFS) fitting was applied to extract quantitative structural results for the Rh moiety in Rh_1_/AC-SNI. The peaks at 2.35 and 2.66 Å correspond to Rh-S and Rh-I coordination in the fresh Rh_1_/AC-SNI, with coordination numbers of 0.7 and 2.6, respectively. The Rh–C/N bonds are assigned as 2.07 Å in the process of fitting, and their coordination number is 1.4 (Fig. 1f, Supplementary Fig. 8, and Supplementary Table 2). To build a rational structure of the catalyst, we performed CO temperature-programmed desorption mass spectrometry (CO-TPD MS) experiments. It can be found that the molar ratio of CO/Rh for Rh_1_/AC-SNI, Rh_1_/AC-NI, and Rh_1_/AC-SI are 1.06, 1.04, and 1.94, respectively, suggesting the coordination number of Rh-CO on Rh_1_/AC-SNI, Rh_1_/AC-NI, and Rh_1_/AC-SI were 1, 1, and 2, respectively^39,40^ (Supplementary Fig. 9). Combined results from XAFS and CO-TPD MS, we can conclude that the Rh_1_ on Rh_1_/AC-SNI mainly exists in the form of Rh(CO)I_3_(N-AC)(S-AC). S-AC and N-AC denoted the sulfur and nitrogen-containing groups. Similarly, Rh(CO)_2_I_2_(S-AC)(O-AC) and Rh(CO)I_3_(N-AC)(O-AC) are the main molecular structure of Rh_1_/AC-SI and Rh_1_/AC-NI, respectively. In accordance with the XANES results, the X-ray photoelectron spectroscopy (XPS) of Rh 3*d* shows the coexistence of Rh^1+^ and Rh^3+^ species on Rh_1_/AC-SNI, Rh_1_/AC-NI, and Rh_1_/AC-SI catalysts (Supplementary Fig. 10 and Supplementary Table 3). Additionally, Maria and Ma Ding et al. believe that CO is an important ligand that can maintain the valence state of metals^6,24^. We designed quasi-in situ XPS to analyze the role of CO on Rh_1_/AC-SNI during the reaction. The sample was pretreated in a mixture of C_2_H_6_, H_2_O, and O_2_ at 423 K for 2 h. The binding energy of Rh 3*d* 5/2 overall shifted to a higher value at 311.6 eV (Supplementary Fig. 11 and Supplementary Table 3). This indicates that CO plays a role in maintaining the valence state of Rh during the reaction, ensuring the optimal coordination environment of active sites. As shown in Supplementary Figs. 12–13 and Supplementary Table 4, comparing with the N species on the AC-SNI carrier, it was found that a new peak appeared at 399.2 eV after Rh loaded on Rh_1_/AC-SNI, confirming the presence of Rh–N coordination^41–43^. Simultaneously, the binding energy of C–S–C at fresh Rh_1_/AC-SNI shifted to low energy direction about 0.4 eV (Supplementary Fig. 14 and Supplementary Table 5), in contrast to that of AC-SNI, suggesting that the S of the C–S–C species withdrew electrons from Rh, causing increase of the electron cloud density around S. Furthermore, we calculated the Bard charges of Rh on Rh_1_/AC-SNI, Rh_1_/AC-SI, and Rh_1_/AC-NI catalysts and found that the chemical state of Rh_1_/AC-SNI was the highest (Supplementary Fig. 15), consistent with the XPS and XANES experimental results.

### Catalyst evaluation

**Fig. 2:**
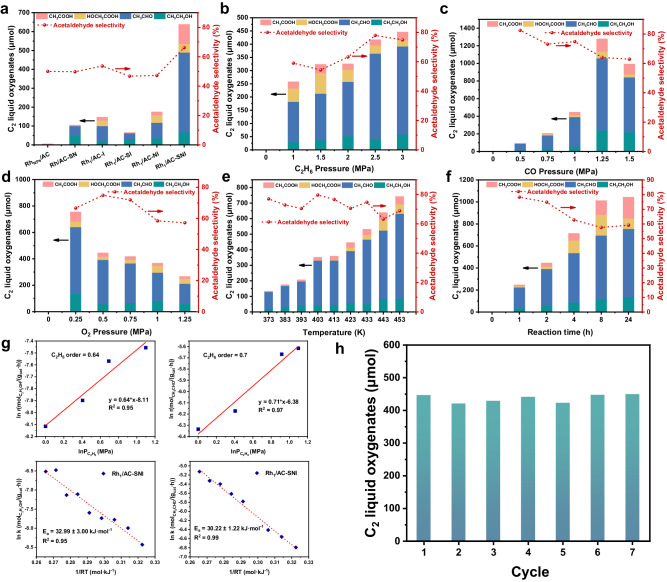
Ethane oxidation performance and structure-activity relationship. Catalytic activity of **a** different catalysts. **b** Different pressure of C_2_H_6_. **c** Different pressure of CO. **d** Different pressure of O_2_. **e** Different reaction temperatures. **f** Different reaction time. **g** Reaction kinetic test on Rh_1_/AC-SNI for ethane direct oxidation. Glycolic acid is included in the ethanol pathway and acetic acid is included in the acetaldehyde pathway. **h** Recyclability tests of Rh_1_/AC-SNI (general reaction conditions: *T* = 423 K, ${P}_{{C}_{2}{H}_{6}}\,$ = 3.0 MPa, ${P}_{{CO}}$ = 1.0 MPa, and ${P}_{{O}_{2}}\,$ = 0.5 MPa over Rh_1_/AC-SNI for 2 h, *m* (H_2_O) = 10 g, *m* (catalyst) = 50 mg).

A typical experiment was carried out in a 100 mL batch reactor containing a polytetrafluoroethylene (PTFE) lining by adding 50 mg catalyst to 10 g deionized water under the conditions of 30 bar C_2_H_6_, 1 bar CO, and 0.5 bar O_2_ at 423 K. The catalytic performance for the oxidation of ethane was measured over these catalysts. For comparison, the Rh_NPs_/AC catalyst results in a higher activity than those of Rh/SiO_2_ and Rh/CeO_2_ (Supplementary Figs. 16 and 17). Nonetheless, it is still inferior to that of the series of Rh_1_/AC-x catalysts (Fig. 2a). Moreover, the activity test shows that Rh_1_/AC-SNI is superior to that Rh_1_/AC-SN, Rh_1_/AC-I, Rh_1_/AC-SI, and Rh_1_/AC-NI under the same condition, demonstrating the positive effect of nitrogen and sulfur species on ethane oxidation (Fig. 2a and Supplementary Table 6).

With the increase of the partial pressure of C_2_H_6_ from atmospheric pressure to 3.0 MPa in Fig. 2b, the TOF of total C_2_ oxygenated products steadily increased. Along with the partial pressure of CO increase from atmospheric pressure to 1.5 MPa, the TOF of total C_2_ oxygenates products reached the maximum value of 158.5 h^−1^ and the ethane conversion is 1.98% at 1.25 MPa (Fig. 2c). An excess of CO concertation will reduce the activity. Similarly, when the partial pressure of O_2_ is 0.25 MPa, the TOF of oxygenated compounds reaches a maximum (Fig. 2d), which indicates that higher oxygen pressure will cause over-oxidation of the product to CO_2_ and keeping the ratio of CO/O_2_ greater than 2.0 will result in a higher TOF. By performing parallel experiments to remove any one of C_2_H_6_, CO, or O_2_, none of the studies showed the production of ethanol, acetaldehyde, acetic acid, and other products, suggesting that the participation of all three gases is indispensable for the formation of C_2_ oxygenates. When the temperature increased from 373 to 453 K, the conversion of ethane and TOF of total C_2_ oxygenates products both increased significantly. As the main product, the selectivity of acetaldehyde remains basically unchanged at about 70%. The TOF of Rh_1_/AC-SNI can reach 34.5 h^−1^ at 373 K (Fig. 2e), greatly exceeding 7.5 h^−^^1^ on Ir-based catalysts by Martin^24^ under the same temperature condition. With the prolonged reaction time, the TOF of total C_2_ oxygenates products also increased until 8 h and then remained unchanged (Fig. 2f). The reaction order of ethane to generate ethanol on Rh_1_/AC-SNI is 0.6 with the reaction activation energy of 32.99 kJ mol^−1^, and the reaction order of generating acetaldehyde is 0.7 with the reaction activation energy of 30.22 kJ mol^−1^, which is consistent with the higher acetaldehyde selectivity (Fig. 2g). In addition, the stability of Rh_1_/AC-SNI catalyst was verified with seven reaction cycles, showing ignorable activity decay at 423 K, 4.5 MPa total pressure and 2 h reaction time (Fig. 2h). It is worth noting, that the highest TOF of C_2_ oxygenates on Rh_1_/AC-SNI came up to 158.5 h^−1^ at 423 K, higher than those of previous reports on direct conversion of ethane at low-temperature^21,24^. Meanwhile, the activity of Rh_1_/AC-SNI was also obviously higher than those of Mo, Pt, Pd, and Ir supported on AC-SNI with the same loading, the highest activity of which is only one-sixth of Rh_1_/AC-SNI under the same reaction conditions (Supplementary Fig. 18 and Supplementary Table 7). In addition, it is found that the microstructure of the Rh_1_/AC-SNI-spent catalyst did not change significantly after the reaction (Supplementary Figs. 2e, 6d, and 7d) and the coordination environment remained basically the same comparing the AC-HADDF-STEM and EXAFS characterization results (Supplementary Figs. 19–21), indicating the stable coordination ability of Rh_1_/AC-SNI.

**Fig. 3:**
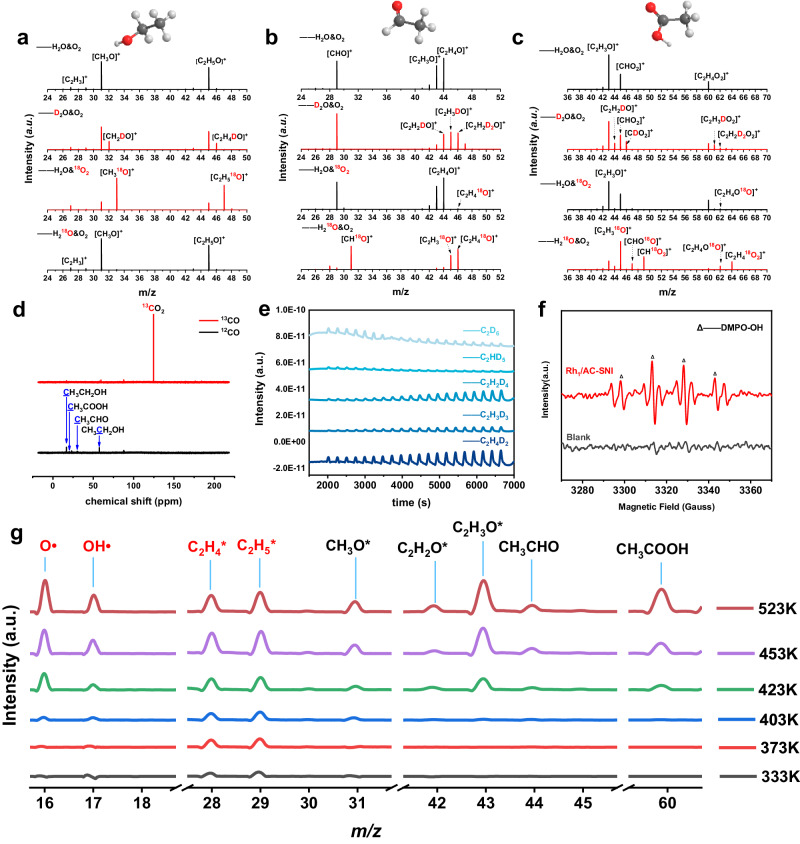
In situ experiments. **a** Ethanol, **b** acetaldehyde, and **c** acetic acid GC–MS spectra, respectively with H_2_O/D_2_O/^18^O_2_/H_2_^18^O isotope labeled on Rh_1_/AC-SNI catalyst. **d** Liquid-phase ^13^C NMR product spectra of CO and ^13^CO as reactants. **e** C_2_H_6_–D_2_-TPD-MS experiment on Rh_1_/AC-SNI catalyst. **f** EPR spectra of the ethane oxidation reaction over Rh_1_/AC-SNI. DMPO was added to the reaction mixture as the radical trapping agent. **g** In situ FEL-TOF/MS spectrometry on Rh_1_/AC-SNI using C_2_H_6_/CO/O_2_/H_2_O/Ar at 303–523 K.

For the isotope labeling experiments on Rh_1_/AC-SNI catalyst in Fig. 3a–c, the mass fragment groups of ethanol, acetaldehyde, and acetic acid were CH_3_O and C_2_H_5_O, CHO, C_2_H_3_O, and C_2_H_4_O, C_2_H_3_O, CHO_2_ as well as C_2_H_4_O_2_ species (Supplementary Tables 8–10). Only one D-atom exchange occurred in ethanol product (Fig. 3a), while multiple D-atoms exchange was found in acetaldehyde (Fig. 3b, c), suggesting that the ethyl group only participated in the formation of ethanol while vinyl or its further dehydrogenation intermediates were involved in the formation of acetaldehyde. To identify the carbon source of C_2_ oxygenates, the ^13^CO isotope labeling experiment was carried out on the Rh_1_/AC-SNI catalyst. The signal of ^13^CO_2_ (125.25 ppm) could be clearly observed. In contrast, no signal of ^13^C appeared in C_2_ oxygenates (Fig. 3d), indicating that CO did not directly participate in the reaction but worked as a co-catalyst. And C_2_H_6_ was the only carbon source of the C_2_ oxygenates. To verify the intermediate species of ethane dehydrogenation, we conducted the C_2_H_6_–D_2_-TPD-MS pulse experiment on Rh_1_/AC-SNI (Fig. 3e). A series of C_2_H_6_–D_2_ exchange products on the Rh_1_/AC-SNI catalyst at 423 K, and C_2_H_5_D (*m*/*z* = 31), C_2_H_4_D_2_ (*m*/*z* = 32), C_2_H_3_D_3_ (*m*/*z* = 33), C_2_H_2_D_4_ (*m*/*z* = 34), C_2_HD_5_ (*m*/*z* = 35), and C_2_D_6_ (*m*/*z* = 36) can be observed. Since the mass spectrometer uses an electron impact ionization source (EI source) to detect the intermediates, the signal at *m*/*z* = 31 contains a part of species aroused by ethane isotope natural abundance. Nevertheless, it can be observed that the intensity of *m*/*z* = 31 species quickly reinforced as the number increase of ethane pulse as shown in Supplementary Fig. 22, indicating the formation of C_2_H_5_D species except for the trace natural isotope. In addition, the signal increase of C_2_H_4_D_2_ among these species is the most remarkable with the increase of ethane pulse times (Fig. 3e). These results indicated that multiple C–H bonds of ethane can be simultaneously activated on Rh_1_/AC-SNI and dissociated into different dehydrogenation species, participating in different reaction pathways, respectively.

We also performed isotopic experiments using ^18^O-labeled oxygen and analyzed the liquid-phase product for the Rh_1_/AC-SNI catalyst. A large amount of C_2_H_5_^18^O (*m*/*z* = 47) was generated, showing that ethanol product was basically marked with ^18^O (Fig. 3a). Surprisingly, almost none of ^18^O was detected in acetaldehyde (Fig. 3b, c), indicating that O_2_ only participated in the formation of ethanol. To further explore the oxygen sources of acetaldehyde, we used ^18^O-labeled water for isotope experiments (Supplementary Tables 8–10). Figure 3a shows that none of the ethanol was labeled by ^18^O from water. While all CH^18^O (*m*/*z* = 31), C_2_H_3_^18^O (*m*/*z* = 45), and C_2_H_4_^18^O (*m*/*z* = 46) fragments from acetaldehyde were labeled by ^18^O from water (Fig. 3b). As shown in Fig. 3c, most of the O in acetic acid originated from H_2_^18^O, verified by a large amount of CH^18^O_2_ (*m*/*z* = 49) and C_2_H_4_^18^O_2_ (*m*/*z* = 64). Meanwhile, a small amount of oxygen in acetic acid also came from O_2_, forming CHO^18^O (*m*/*z* = 47) and C_2_H_4_O^18^O (*m*/*z* = 62), indicating that only a small part of acetic acid derived from the oxidation of ethanol by O_2_. Additionally, we explored the universality of the reaction mechanism using Rh-ZSM-5 catalyst. The catalytic activity and ^18^O-GC–MS results are shown in Supplementary Figs. 23 and 24. Despite of the higher acetic acid selectivity of Rh-ZSM-5, the ^18^O distribution of oxygenates for Rh-ZSM-5 is basically consistent with that of Rh_1_/AC-SNI, indicating that the low-temperature direct oxidation reaction mechanism of ethane proposed here has a certain universality. Oxygen exchange between H_2_O and O_2_ is negligible (Supplementary Fig. 25)^44–48^. Isotopic experiments indicated that H_2_O directly participated in the reaction process and provided an oxygen source for acetaldehyde. To explore the reaction routes, ethanol and acetaldehyde were used as a substrate to substitute C_2_H_6_, respectively. The formation rate of acetaldehyde and acetic acid was very slow using ethanol substrate. However, acetaldehyde substrate was quickly converted into acetic acid, suggesting that the oxidation energy barrier of ethanol was much higher than that of acetaldehyde to form acetic acid on Rh_1_/AC-SNI catalyst (Supplementary Fig. 26). Electron paramagnetic resonance (EPR) with 5,5-dimethyl-1-pyrroline N-oxide (DMPO) as the radical scavenger was conducted^45,49^. Figure 3f showed that hydroxyl radicals (•OH) decomposed from H_2_O during the reaction process can be captured by DMPO in the liquid phase with clear response signals, which directly participated in the reaction with ethyl or ethylidene on Rh_1_/AC-SNI catalyst.

In situ free-electron laser time of flight mass spectrometry (FEL-TOF/MS) using the vacuum ultraviolet free-electron laser (VUV FEL) at the Dalian Coherent Light Source of China (Supplementary Fig. 27)^50,51^. Some key intermediate species, such as oxygen radicals (•O), hydroxyl radicals (•OH), vinyl groups (C_2_H_4_*), and ethyl groups (C_2_H_5_*) were captured when C_2_H_6_/CO/O_2_/H_2_O/Ar mixture passed through Rh_1_/AC-SNI (Fig. 3g and Supplementary Fig. 28). Meanwhile, we also observed the obvious signals of CH_3_O*, C_2_H_2_O*, C_2_H_3_O*, acetaldehyde, acetic acid, and other products and their corresponding fragmentation species at 523 K. The signal intensity of the intermediate species gradually strengthened with the temperature increase. The signals of •O (*m*/*z* = 16), •OH (*m*/*z* = 17), acetaldehyde, and acetic acid started to be obvious at 423 K, showing that the splitting of oxygen and water in the reaction system needs a minimum activation temperature. It can be concluded that oxygen and water both provided an O source for C_2_ oxygenates in the reaction. Oxygen and ethyl groups from ethane played a determining role in the formation of ethanol. Correspondingly, water and vinyl intermediates decided the generation of acetaldehyde. The generation pathways of ethanol and acetaldehyde should follow two different pathways, also proposed by Pan et al. in the process of ethane oxidation by in situ synchrotron radiation photoionization mass spectrometry^51^.

**Fig. 4:**
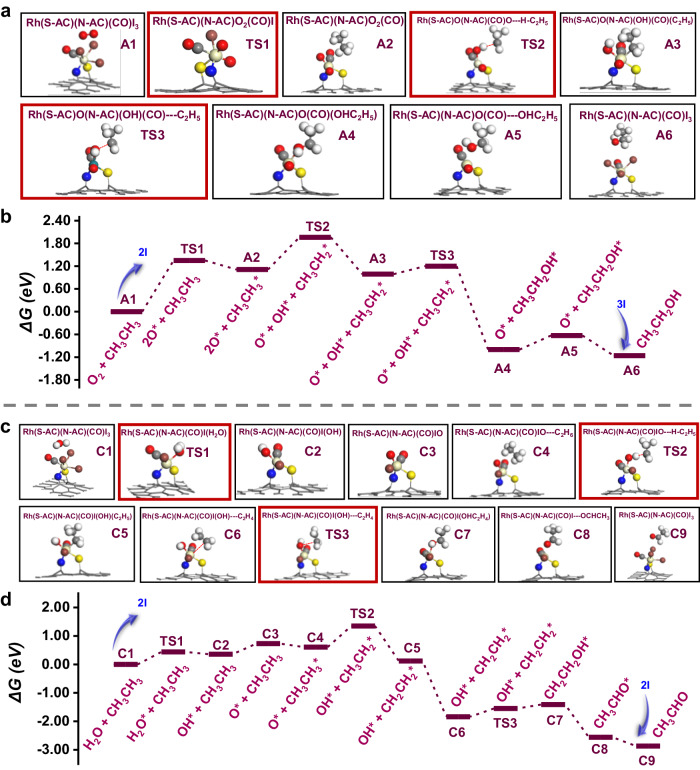
DFT calculations. **a** Structures of the key intermediates involved in the reaction pathway of C_2_H_6_ and O_2_ to ethanol on Rh_1_/AC-SNI catalyst. **b** The free energy (Δ*G*) diagrams of the reaction pathway of O_2_ participating in the production of ethanol. The states A1–A6 represent different basic states in the reaction pathway, and TS represents the transition state. **c** Structures of key intermediates involved in the reaction pathway of C_2_H_6_ and H_2_O to acetaldehyde on Rh_1_/AC-SNI catalyst. **d** The free energy (Δ*G*) diagrams of the reaction pathway of H_2_O participating in the production of acetaldehyde. States C1–C9 represent different basic states in the reaction pathway, and TS represents the transition state. Colors in the picture: the white balls are H; the gray balls are C; the red balls are O; the blue balls are N; the yellow balls are S; the brown balls are I; the beige balls are Rh.

Density functional theory (DFT) calculations were used to simulate the reaction path and energy change. In the path of ethanol formation (Fig. 4a, b), the O radical from O_2_ would replace two I atoms in the initial Rh_1_(N-AC)(S-AC)(CO)I_3_ (**A1**) species and generate the transition state Rh_1_–2O* (**TS1**) with an activation barrier of 1.35 eV as the rate-determining step. Rh_1_–2O* can activate C_2_H_6_ to C_2_H_5_* and generate •OH to form Rh_1_–(O*)(OH*)(C_2_H_5_*) (**TS2**) with the attack of ethane. Then the ethyl group migrates from the Rh_1_–(O*)(OH*) active site to the hydroxyl group with an energy barrier of 0.21 eV to form Rh_1_–(O*)(CH_3_CH_2_OH*) (**TS3**). Ethanol species are rapidly released into the solution without any energy barrier. Interestingly, when the •OH provided by H_2_O is used as the active O species, the energy barrier for ethane to break the C–H bond is as high as 3.33 eV (Supplementary Fig. 29-TS2), which indicates that O_2_ can activate ethane molecules more effectively in the ethanol formation pathway. Philippe et al. found that alkane-to-metal donation determines the stability of the metal-alkane complex and metal-to-alkane back-donation facilitates C–H bond cleavage by oxidative addition in the σ-complexes^52^. Herein, we activated the C–H bond by constructing similar σ-complexes and further transformed it into different oxygenates.

**Fig. 5:**
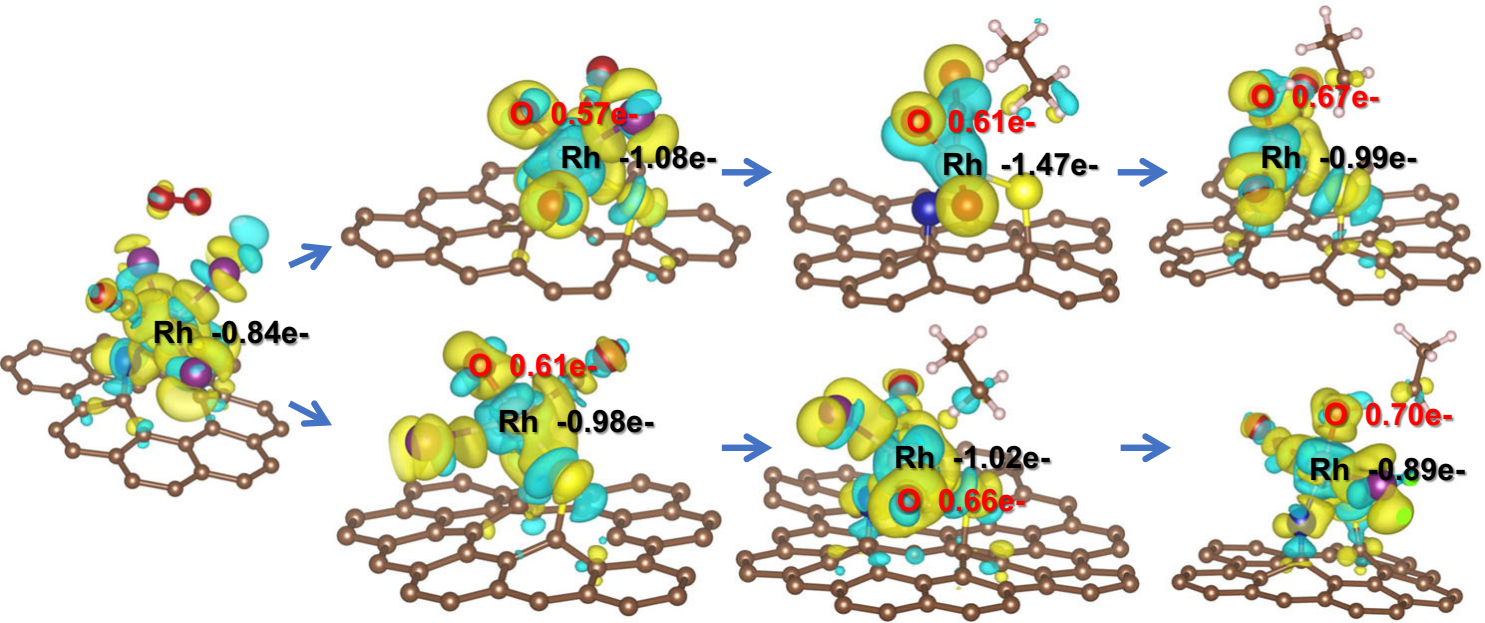
DFT calculations. The differential charge density on the Rh active site in both pathways in units of negative charge e^−^. The Bader charge change values for Rh and O are marked. For the differential charge density, the cyan areas are electron deficient and the yellow areas are electron rich.

For the path of acetaldehyde generation (Fig. 4c, d), the H_2_O molecule also replaced two I atoms of the same initial active site Rh_1_(N-AC)(S-AC)(CO)I_3_ (**C1**) to sequentially form Rh_1_–(OH*) (**C2**) via the transition state Rh_1_–H_2_O (**TS1**). **C2** was further oxidized by O_2_ to form Rh_1_–O* (**C3**). Ethane near Rh_1_–O* will be activated to C_2_H_5_ and adsorbed on the Rh_1_ active site to form Rh_1_–OH*–C_2_H_5_*(**TS2**) as the rate-determining step with an activation barrier of 0.74 eV. C_2_H_5_* will be oxidized to vinyl by active •O in water and instantly bond with OH* via the **TS3** transition state to form Rh–C_2_H_4_OH*(C7). C_2_H_4_OH* is extremely prone to reconstitution. Then the formed acetaldehyde detaches from the Rh active site. Nevertheless, the energy barrier of the rate-determining step is as high as 1.35 eV (Supplementary Fig. 30-TS1) when oxygen participates in the formation pathway of acetaldehyde, indicating that H_2_O is a more efficient oxidant in the formation pathway of acetaldehyde. Moreover, the energy barrier of the rate-determining step for acetaldehyde formation was 0.74 eV, much lower than 1.35 eV of ethanol formation, consistent with the higher selectivity of acetaldehyde in all the products. The Bader charge shows that the electron cloud density around O gradually increases in both paths (**A1-TS2** and **C1-TS2**), and the nucleophilicity of O increases, which is conducive to capturing H on C_2_H_6_ (Fig. 5). Before O captures H, the electron cloud around Rh gradually decreases, while the Rh–O bond interaction becomes weaker, and the electron cloud density of Rh increases after the σ-bond in O–H is formed, which is still conducive to the nucleophilic attack of the ethyl group.

We also compared the energy barriers of ethane with O_2_ to ethanol over the Rh_1_/AC-SI and Rh_1_/AC-NI catalysts as shown in Supplementary Figs. 31 and 32. The energy barrier of the rate-determining step follows in order of Rh_1_/AC-SI > Rh_1_/AC-NI > Rh_1_/AC-SNI (Supplementary Fig. 33 and Supplementary Tables 11, 15, and 16), which is also consistent with their corresponding catalytic activity (Fig. 2a).

## Discussion

In summary, we have successfully synthesized a series of Rh_1_ catalysts supported on S, N, and I co-doped AC. The coordination environment of Rh_1_ was modulated by the functional groups in the carbon matrix, as proved by various experimental characterizations. Among these catalysts, Rh_1_/AC-SNI exhibited the highest C_2_ oxygenates TOF of 158.5 h^−1^ for the selective oxidation of ethane at low-temperature, obviously higher than those of Rh_1_/AC-NI, Rh_1_/AC-SI, Rh_1_/AC-SN, Rh_1_ /AC-I, and Rh_NPs_/AC catalysts under the same mild reaction conditions. Acetaldehyde was the main product in all the C_2_ oxygenates. The formation mechanism of acetaldehyde and ethanol followed two independent pathways, verified by an ^18^O isotope labeling experiment. The oxygen source of ethanol and acetaldehyde is derived from oxygen and water, respectively. Oxygen radicals (•O), hydroxyl radicals (•OH), vinyl groups (C_2_H_4_*), and ethyl groups (C_2_H_5_*) key intermediates were in situ captured by in situ FEL-TOF/MS. Density function theoretical calculations also demonstrated the different reaction pathways for the generation of ethanol and acetaldehyde on Rh_1_/AC-SNI catalyst, showing that the energy barrier of the rate-determining step for acetaldehyde production was 0.74 eV, lower than 1.35 eV of ethanol formation.

## Methods

### Preparation of S, N-doped activated carbon (AC/SN)

For the synthesis of AC-SN, 1.0 g AC, and 1.0 g thioacetamide (C_2_H_5_NS) were mixed well, then the uniformly mixed powder was heated to 673 K with a heating rate of 1 K min^−1^ under a flowing Ar (30 mL min^−^^1^) atmosphere for 2 h. After cooling to room temperature, the resulting samples were washed with ultra-pure water three times and then dried at 393 K to get the AC-SN support. For the preparation of N-doped activated carbon (AC-N) and S-doped activated carbon (AC-S), the synthesis protocol was similar to that of the AC-SN except that the precursor melamine (C_3_H_6_N_6_) and benzyl disulfide (C_14_H_14_S_2_) were used as the N and S sources, respectively.

### Preparation of catalysts

The AC-SN was pretreated with CH_3_I in a quartz tube furnace at 513 K for 4 h, and CH_3_I was bubbled by N_2_ flow (50 mL min^−1^) into the tube furnace. The as-prepared sample was denoted as AC-SNI. For comparison, pure AC, AC-N, and AC-S samples were treated with the same procedure, denoted as AC-I, AC-NI, and AC-SI, respectively. Subsequently, a certain amount of Rh_2_(CO)_4_Cl_2_ was dissolved in CH_2_Cl_2_ (15 mL), and the 1.0 g AC-SNI was added into the solution for 24 h stirring at room temperature. The final product was filtered and washed with CH_2_Cl_2_ at least three times and dried at 333 K overnight to get the Rh_1_/AC-SNI catalyst. Similarly, Rh_1_/AC-I, Rh_1_/AC-SN, Rh_1_/AC-NI, and Rh_1_/AC-SI samples were also prepared with the same method using AC-I, AC-SN, AC-NI, and AC-SI supports. Rh/SiO_2_ and Rh/CeO_2_ were prepared by traditional impregnation method using RhCl_3_ aqueous solution and then calcined at 673 K for 2 h under Ar atmosphere. The Rh_NPs_/AC catalyst was prepared by impregnation of Rh_2_(CO)_4_Cl_2_ solution in dichloromethane with pure AC and then calcined at 673 K for 2 h under an Ar atmosphere. The theoretical loading of Rh for all the catalysts was 1 wt%.

### Catalyst performance evaluation

The direct oxidation of ethane was carried out in a 100 mL batch reactor containing a PTFE lining. For a typical experiment, 50 mg catalyst and 10 g water were added, followed by purging the reactor five times with 2.0 MPa ethane (99.999%). Three megapascal ethane was then added at room temperature firstly, followed by 1.0 MPa CO and 0.5 MPa oxygen. After no variation in pressure, the autoclave was heated to the reaction temperature at a rotational speed of 600 rpm and maintained for a certain time. After the reaction, the reactor was rapidly moved into an ice bath and lowered to below 283 K. The reaction time refers to the thermostatic period. The gas phase products analysis was performed on Agilent Technologies 7890B gas chromatography system using a TDX-01 packed column. The liquid-phase product was obtained by suction filtration of the obtained liquid-phase mixture. Two-hundredths percentage by weight DSS solution was prepared by dissolving 4,4-dimethyl-4-silapentane-1-sulfonic acid (DSS) in deuterium oxide (D_2_O) as the internal standard. A linear relationship between the peak areas ratio of oxygenates to DSS was used to establish a standard curve, setting the DSS chemical shift as δ = 0. 1400 μL liquid product and 200 μL DSS standard solution were mixed up by ultrasonic for 10 min. Eight hundred microliter of the mixed liquid was extracted for the ^1^H-NMR test. ^1^H NMR was collected on a Bruker 700 MHz spectrometer. According to the qualitative analysis method by NMR results, the chemical shifts at δ = 1.17, 3.65 ppm were attributed to CH_3_CH_2_OH; the chemical shifts at δ = 1.33, 2.23, 9.67 ppm were attributed to CH_3_CHO, and the chemical shift at δ = 2.08 ppm was attributed to CH_3_COOH. Each data was essentially repeated over three times under the same terms. The carbon balance was between 95% and 105% for the catalytic tests conducted. The acetaldehyde selectivity (%), and TOF were calculated as following Eqs. (1–2):

1 $${{{{{\rm{acetaldehyde}}}}}}\; {{{{{\rm{selectivity}}}}}}=\frac{{{\it{n}}}_{{{{{{\rm{(acetaldehyde)}}}}}}}}{{{\it{n}}}_{{{{{{\rm{(oxygenates)}}}}}}}}\times 100\%$$

2 $${{{{{\rm{TOF}}}}}}=\frac{{{\it{n}}}_{{{{{{\rm{(oxygenates)}}}}}}}}{{{\it{n}}}_{{{{{{\rm{(Rh)}}}}}}}\times {\it{t}}}$$

### Catalyst characterization

SEM images were obtained with a Quanta 400 FEG instrument at an accelerating voltage of 0.01–30 kV. A Quanta 400 FEG instrument was used to obtain SEM images at an acceleration voltage of 0.01–30 kV. HR-TEM images were taken with a TECNAI G2 F30 instrument at an accelerating voltage of 200 kV. The high-angle circular dark-field scanning transmission electron microscope (HAADF-STEM) and EDS mapping images were obtained on a JEM-ARM200F instrument with a CEOS probe corrector working at 200 kV to guarantee a resolution of 0.08 nm. The Quantachrome Autosorb-1 system was used to determine the nitrogen sorption isotherms at the temperature of liquid nitrogen and the samples were outgassed for 12 h at 393 K before the measurements. The pore size distribution curves of the adsorbed branch were determined by the quenched solid density functional theory (QSDFT) method. Within the relative pressure *P*/*P*_0_ = 0.3, the specific surface area was calculated from the adsorption data using the Brunauer–Emmett–Teller (BET) method. The pore size distribution of AC and the catalyst was mainly concentrated in the micropore range (<2 nm) calculated from the QSDFT adsorption method. XRD measurements were performed using a PANalytical X’Pert Pro X-ray diffractometer with a Cu Kα X-ray source at a wavelength of 1.5045 Å. CO-TPD MS of Rh_1_/AC-SNI was performed by an AMI-300 chemisorption analyzer. Sixty milligram sample was first treated for 1 h at 303 K in a flow of He (30 mL min^−1^) to remove physically adsorbed substances. After the baseline stabilized, TPD data was collected using a thermal conductivity detector (TCD) at a heating rate from 10 K min^−1^ to 1273 K. To quantify the total desorbed CO, the peak area of the TCD signal was calibrated by pulse sampling using a 10% CO/He standard gas. XPS was performed with a Thermo Fisher Scientific ESCALAB 250Xi instrument under Al Kα irradiation, and the binding energy was calibrated with reference to the C 1 s peak (284.8 eV). The inductively coupled plasma emission spectrometer (ICP-OES) analysis was performed on the PerkinElmer Optima 7300 DV instrument, and the powder samples were pretreated on the Anton Paar Multiwave 3000 system.

The C_2_H_6_–D_2_-TPD-MS experiment was carried out on an Autochem II 2920 instrument. D_2_ was used as the carrier gas and the flow rate was 15 mL min^−1^. Ethane (99%) was used as the pulse gas. Fifty milligram sample was firstly pretreated at 373 K under an Ar atmosphere for 30 min and then switched to a D_2_ atmosphere. After the baseline was stabilized, the temperature was raised to 423 K and held for 1 h with 20 pulses of ethane, and meanwhile monitored by mass spectrometry. The EPR spectrums were obtained on a Bruker A200 instrument with a microwave frequency of 9.3246 GHz. The reaction autoclave was quickly transferred to an ice bath and cooled to below 278 K after the reaction, then the liquid phase product was extracted and quickly transferred to a cold tank stored below 273 K by liquid nitrogen. The gas chromatography-mass spectrometry (GC–MS) experiment was carried out on a gas chromatography linked with quadrupole battle-time high-resolution mass spectrometer (Agilent Technologies Inc 8890-7250), which was equipped with a DB-WAX column and two 5A mol sieve column, TCD, and flame ionization detector (FID). The column temperature was kept at 313 K for 5 min, then increased to 523 K at a rate of 10 K min^−1^, and then kept at 523 K for 10 min.

The short-lived intermediate species can be observed via in situ FEL-TOF/MS experiments were conducted to detect the in situ formed short-lived radical species. For in situ experiments, the Rh_1_/AC-SNI sample was placed at the back end of the quartz tube reactor. The rear end was designed as an inverted cone with an extremely small hole at the apex for the diffusion of reactants and radical species into the detection chamber, and the back end of the quartz tube was wrapped with resistance wire to heat the reaction tube. After introducing C_2_H_6_/CO/O_2_/H_2_O/Ar into the reactor, the pressure of the reactor and chamber was kept at 10^-2^ and 10^-7 ^Torr. H_2_O steam was carried in through C_2_H_6_/CO/ Ar bubbling at ambient temperature. Subsequently, the Rh_1_/AC-SNI sample was heated from room temperature to the test temperature in the C_2_H_6_/CO/O_2_/H_2_O/Ar mixture flow, (C_2_H_6_/CO = 1:1, 5 mL min^−1^; O_2_/Ar = 1:1, 5 mL min^−1^). Substances in the detection chamber were ionized by the VUV FEL and the resulting ions were analyzed by time-of-flight mass spectrometry equipped with a microchannel plate detector. In this work, the pulse energy was 8 ~ 9 μJ/pulse operating at 10 Hz, and the output wavelength was continuously 115 nm. The EXAFS spectrum of the catalyst was tested on the edge of Rh K using the Si (3 1 1) crystal monochromator at the BL14W1 beamline of SSRF of SINAP (Shanghai, China). The storage ring operated at 3.5 GeV and the injection current was 200 mA. Rh foil was used as a reference sample, and all X-ray absorption spectra were obtained in fluorescence mode.

### Density functional theory (DFT) calculations

All the DFT calculations were performed using Vienna Ab-initio Simulation Package (VASP) code^53,54^ with electron correction treated within the generalized gradient approximation using the Perdew–Burke–Ernzerhof (PBE) exchange-correlation functional^55,56^. The projector augmented wave (PAW) method^57^ was used to treat the effect of the inner cores on the valence states. The slab of Rh anchored on the N/S co-doped carbon materials was set. The reaction on the surface of single-Rh-sites (co-doped C (001)) was carried out using the slab models composed of p(7 × 7) supercells with a single layer. In all the calculations, the cutoff energy was set to be 500 eV and the Gaussian electron smearing method with σ = 0.05 eV was used. The convergence tolerance for residual force and energy on each atom during structure relaxation was set to 0.05 eV/Å and 10^−5^ eV, respectively. The Monkhorst–Pack grids^58^ were set to be 4 × 4 × 1 surface optimizations. A vacuum layer of 20 Å along the *z* direction was introduced to eliminate the spurious interactions between adjacent sheets.

### Supplementary information

Supplementary Information

Peer Review File

## Data Availability

All data supporting the findings of this study are available within the paper and its Supplementary Information files.

## References

[1] Periana RA, Mironov O, Taube D, Bhalla G, Jones CJ (2003). Catalytic, oxidative condensation of CH_4_ to CH_3_COOH in one step via CH activation. Science.

[2] Hashiguchi BG (2014). Main-group compounds selectively oxidize mixtures of methane, ethane, and propane to alcohol esters. Science.

[3] Yuan Q, Deng W, Zhang Q, Wang Y (2007). Osmium-catalyzed selective oxidations of methane and ethane with hydrogen peroxide in aqueous medium. Adv. Synth. Catal..

[4] Wang S (2019). Room-temperature conversion of ethane and the mechanism understanding over single iron atoms confined in graphene. J. Energy Chem..

[5] Tang Y (2018). Single rhodium atoms anchored in micropores for efficient transformation of methane under mild conditions. Nat. Commun..

[6] Shan JJ, Li MW, Allard LF, Lee SS, Flytzani-Stephanopoulos M (2017). Mild oxidation of methane to methanol or acetic acid on supported isolated rhodium catalysts. Nature.

[7] Tomkins P (2016). Isothermal cyclic conversion of methane into methanol over copper-exchanged zeolite at low temperature. Angew. Chem. Int. Ed. Engl..

[8] Woertink, J. S. et al. A Cu2O (2+) core in Cu-ZSM-5, the active site in the oxidation of methane to methanol. *Proc. Natl. Acad. Sci. USA* **106**, 18908–18913 (2009). 10.1073/pnas.0910461106 PMC2776445 19864626

[9] Lustemberg PG (2018). Direct conversion of methane to methanol on Ni-ceria surfaces: metal-support interactions and water-enabled catalytic conversion by site blocking. J. Am. Chem. Soc..

[10] Liu ZY (2020). Water-promoted interfacial pathways in methane oxidation to methanol on a CeO_2_–Cu_2_O catalyst. Science.

[11] Koishybay A, Shantz DF (2020). Water is the oxygen source for methanol produced in partial oxidation of methane in a flow reactor over Cu-SSZ-13. J. Am. Chem. Soc..

[12] Qi G (2022). Au-ZSM-5 catalyses the selective oxidation of CH_4_ to CH_3_OH and CH_3_COOH using O_2_. Nat. Catal..

[13] Wu B (2022). Fe binuclear sites convert methane to acetic acid with ultrahigh selectivity. Chem.

[14] Groothaert MH, Smeets PJ, Sels BF, Jacobs PA, Schoonheydt RA (2005). Selective oxidation of methane by the Bis(μ-oxo)dicopper core stabilized on ZSM-5 and mordenite zeolites. J. Am. Chem. Soc..

[15] Pacala S, Socolow R (2004). Stabilization wedges: solving the climate problem for the next 50 years with current technologies. Science.

[16] Tang P, Zhu Q, Wu Z, Ma D (2014). Methane activation: the past and future. Energy Environ. Sci..

[17] Rahimi N, Karimzadeh R (2011). Catalytic cracking of hydrocarbons over modified ZSM-5 zeolites to produce light olefins: a review. Appl. Catal. A.

[18] Cossee P (1964). Mechanism of polymerization of alpha-olefins with Ziegler–Natta Catalysts. J. Catal..

[19] Blanksby SJ, Ellison GB (2003). Bond dissociation energies of organic molecules. Acc. Chem. Res..

[20] Armstrong, R., Hutchings, G. & Taylor, S. An overview of recent advances of the catalytic selective oxidation of ethane to oxygenates. *Catalysts* **6**, 71 (2016).

[21] Forde MM (2013). Partial oxidation of ethane to oxygenates using Fe- and Cu-containing ZSM-5. J. Am. Chem. Soc..

[22] Wang Y, Otsuka K (1997). Partial oxidation of ethane by reductively activated oxygen over iron phosphate catalyst. J. Catal..

[23] Wang YL, Gurses S, Felvey N, Kronawitter CX (2020). Room temperature and atmospheric pressure aqueous partial oxidation of ethane to oxygenates over AuPd catalysts. Catal. Sci. Technol..

[24] Jin R (2019). Low temperature oxidation of ethane to oxygenates by oxygen over iridium-cluster catalysts. J. Am. Chem. Soc..

[25] Lin MR, Hogan T, Sen A (1997). A highly catalytic bimetallic system for the low-temperature selective oxidation of methane and lower alkanes with dioxygen as the oxidant. J. Am. Chem. Soc..

[26] Gu, F. B. et al. Selective catalytic oxidation of methane to methanol in aqueous medium over copper cations promoted by atomically dispersed rhodium on TiO_2_. *Angew. Chem. Int. Ed*. **61**, e202201540 (2022). 10.1002/anie.202201540 35199428

[27] Feng S (2019). In situ formation of mononuclear complexes by reaction-induced atomic dispersion of supported noble metal nanoparticles. Nat. Commun..

[28] Qi J (2020). Selective methanol carbonylation to acetic acid on heterogeneous atomically dispersed ReO_4_/SiO_2_ Catalysts. J. Am. Chem. Soc..

[29] Cui X, Li W, Ryabchuk P, Junge K, Beller M (2018). Bridging homogeneous and heterogeneous catalysis by heterogeneous single-metal-site catalysts. Nat. Catal..

[30] Kim YT, Uruga T, Mitani T (2006). Formation of single Pt atoms on thiolated carbon nanotubes using a moderate and large-scale chemical approach. Adv. Mater..

[31] Kumar P (2023). Multifunctional carbon nitride nanoarchitectures for catalysis. Chem. Soc. Rev..

[32] Wang, L. et al. A sulfur-tethering synthesis strategy toward high-loading atomically dispersed noble metal catalysts. *Sci. Adv*. **5**, eaax6322 (2019). 10.1126/sciadv.aax6322 PMC6814374 31692785

[33] Yang CL (2021). Sulfur-anchoring synthesis of platinum intermetallic nanoparticle catalysts for fuel cells. Science.

[34] Feng S (2023). Sulfur-poisoning on Rh NP but sulfur-promotion on single-Rh_1_-site for methanol carbonylation. Appl. Catal. B.

[35] Mu, J. et al. Engineering the coordination environment of single-Rh-site with N and S Atoms for efficient methanol carbonylation. *Adv. Funct. Mater*. **33**, 2305823 (2023).

[36] Li, B. et al. Direct conversion of methane to oxygenates on porous organic polymers supported Rh mononuclear complex catalyst under mild conditions. *Appl. Catal. B* **293**, 120208 (2021).

[37] Wei J (2021). Precisely engineering architectures of Co/C sub-microreactors for selective syngas conversion. Small.

[38] Ryaboshapka, D. et al. Ultradispersed (Co)Mo catalysts with high hydrodesulfurization activity. *Appl. Catal. B* **302**, 120831 (2022).

[39] Li X (2021). Iodide-coordinated single-site Pd catalysts for alkyne dialkoxycarbonylation. ACS Catal..

[40] Ioannides T, Verykios X (1993). Influence of the carrier on the interaction of H_2_ and CO with supported Rh. J. Catal..

[41] Ge X (2020). Metal-organic framework-derived nitrogen-doped cobalt nanocluster inlaid porous carbon as high-efficiency catalyst for advanced potassium-sulfur batteries. ACS Nano.

[42] Fei H (2018). General synthesis and definitive structural identification of MN_4_C_4_ single-atom catalysts with tunable electrocatalytic activities. Nat. Catal..

[43] Yang HB (2018). Atomically dispersed Ni(i) as the active site for electrochemical CO_2_ reduction. Nat. Energy.

[44] Agarwal N (2017). Aqueous Au–Pd colloids catalyze selective CH_4_ oxidation to CH_3_OH with O_2_ under mild conditions. Science.

[45] Song H (2019). Direct and selective photocatalytic oxidation of CH_4_ to oxygenates with O_2_ on cocatalysts/ZnO at room temperature in water. J. Am. Chem. Soc..

[46] Song, H. et al. Selective photo-oxidation of methane to methanol with oxygen over dual-cocatalyst-modified titanium dioxide. *ACS Catal*. **10**, 14318–14326 (2020).

[47] Zhu Y (2021). Highly efficient visible-light photocatalytic ethane oxidation into ethyl hydroperoxide as a radical reservoir. Chem. Sci..

[48] Zhang, H. et al. Activation of light alkanes at room temperature and ambient pressure. *Nat. Catal*. **6**, 666–675 (2023).

[49] Hammond C (2012). Direct catalytic conversion of methane to methanol in an aqueous medium by using copper-promoted Fe-ZSM-5. Angew. Chem. Int. Ed. Engl..

[50] Luo L (2019). Gas-phase reaction network of Li/MgO-catalyzed oxidative coupling of methane and oxidative dehydrogenation of ethane. ACS Catal..

[51] Liu, C. Y. et al. Illustrating the fate of methyl radical in photocatalytic methane oxidation over Ag–ZnO by in situ synchrotron radiation photoionization mass spectrometry. *Angew. Chem. Int. Ed. Engl.* **62**, e202304352 (2023). 10.1002/anie.202304352 37219500

[52] Jay RM (2023). Tracking C–H activation with orbital resolution. Science.

[53] Kresse G, Hafner J (1993). Ab initio molecular dynamics for liquid metals. Phys. Rev. B Condens. Matter.

[54] Kresse G, Furthmuller J (1996). Efficient iterative schemes for ab initio total-energy calculations using a plane-wave basis set. Phys. Rev. B Condens Matter.

[55] Perdew, J. P. & Wang, Y. Accurate and simple analytic representation of the electron-gas correlation energy. *Phys. Rev. B* **45**, 13244 (1992); erratum **98**, 079904 (2018). 10.1103/physrevb.45.13244 10001404

[56] Hammer B, Hansen LB, Norskov JK (1999). Improved adsorption energetics within density-functional theory using revised Perdew–Burke–Ernzerhof functionals. Phys. Rev. B.

[57] Blochl PE (1994). Projector augmented-wave method. Phys. Rev. B Condens Matter.

[58] Monkhorst HJ, Pack JD (1976). Special points for Brillouin-zone integrations. Phys. Rev. B.

