# Supplementary material for: Water-participated mild oxidation of ethane to acetaldehyde
Source: Nat Commun. 2024 Mar 22;15:2555. doi: 10.1038/s41467-024-46884-7 (PMC10959925; doi:10.1038/s41467-024-46884-7)
Supplement: Supplementary file 1 — Supplementary Information [file 41467_2024_46884_MOESM1_ESM.pdf]

Supplementary Information

Water-Participated Mild Oxidation of Ethane to Acetaldehyde

Bin Li[1,2#], Jiali Mu[1#], Guifa Long[3#], Xiangen Song[1*], Ende Huang[1,2], Siyue Liu[1,2], Yao Wei[4], Fanfei Sun[4], Siquan Feng[1], Qiao Yuan[1,2], Yutong Cai[1,2], Jian Song[1,2], Wenrui Dong[5,6], Weiqing Zhang[5], Xueming Yang[5,7*], Li Yan[1], Yunjie Ding[1,8*]

[1] Dalian National Laboratory for Clean Energy, Dalian Institute of Chemical Physics, Chinese Academy of Sciences, Dalian, China

[2] University of Chinese Academy of Sciences, Beijing, China

[3] Guangxi Key Laboratory of Chemistry and Engineering of Forest Products, School of Chemistry and Chemical Engineering, Guangxi Minzu University, Nanning, 530008, China

[4]Shanghai Synchrotron Radiation Facility, Shanghai Institute of Applied Physics; Shanghai Advanced Research Institute, Chinese Academy of Sciences, Shanghai, China

[5]State Key Laboratory of Molecular Reaction Dynamics, Dalian Institute of Chemical Physics, Chinese Academy of Sciences, 116023 Dalian, China

[6]Hefei National Laboratory, Hefei,230088, China

[7]Department of Chemistry, Southern University of Science and Technology, Shenzhen 518055, China.

[8]State Key Laboratory of catalysis, Dalian Institute of Chemical Physics, Chinese Academy of Sciences, Dalian, China

[#]These authors contributed equally: Bin Li, Jiali Mu, Guifa Long.

[*]Corresponding Authors:

Yunjie Ding (dyj@dicp.ac.cn); Xiangen Song (xiangensong@dicp.ac.cn); Xueming Yang (xmyang@dicp.ac.cn)

Table of contents

Supplementary Figures

Supplementary Figure 1. SEM images 3

Supplementary Figure 2. Pore size distributions and N_2 sorption isotherms 4

Supplementary Figure 3. N_2-TG 5

Supplementary Figure 4. Raman spectrogram 6

Supplementary Figure 5. XRD patterns 7

Supplementary Figure 6. TEM images 8

Supplementary Figure 7. HAADF-TEM images 9

Supplementary Figure 8. Fourier-transformed magnitude of Rh K-edge EXAFS spectra in k and R space 10

Supplementary Figure 9. CO-TPD of Rh_1-SNI/AC 11

Supplementary Figure 10-11. XPS patterns of Rh 3d 12-13

Supplementary Figure 12. XPS patterns of N 1s 14

Supplementary Figure 13. XPS results of N 1s area percent of different N species 15

Supplementary Figure 14. XPS patterns of S 2p 16

Supplementary Figure 15. The Bader charge of Rh_1 17

Supplementary Figure 16. Activity comparison of different NPs catalysts 18

Supplementary Figure 17. TEM images of Rh_{NPs}/AC 19

Supplementary Figure 18. Activity comparison of different active metal 20

Supplementary Figure 19. The experimental curve of k^1 -weight EXAFS spectra in r-space of fresh Rh_1/AC-SNI and spent Rh_1/AC-SNI. C_2H_6-D_2-TPD-MS experiment 21

Supplementary Figure 20. The wavelet transform contour plots of k^1 -weighted χ(k) EXAFS signals of Rh_1/AC-I, Rh_1-SNI/AC-fresh and Rh_1-SNI/AC-spent 22

Supplementary Figure 21. XANES spectra 23

Supplementary Figure 22. C_2H_6-D_2-TPD-MS experiment 24

Supplementary Figure 23. Activity comparison of Rh_1/AC-SNI and Rh-ZSM-5 25

Supplementary Figure 24. GC-MS spectra on Rh-ZSM-5 catalyst 26

Supplementary Figure 25. GC-MS spectra of water 27

Supplementary Figure 26. Comparison of TOR with ethanol and acetaldehyde as substrates 28

Supplementary Figure 27. Schematic diagram of the experimental setup and quartz tube reactor 29

Supplementary Figure 28. The signal change with temperature increase of in situ free-electron laser time of flight mass spectrometry 30
Supplementary Figure 29-30. DFT theoretical study on the conversion pathway of ethane over Rh_1-SNI/AC catalyst 31-32
Supplementary Figure 31-32. DFT theoretical study on the conversion pathway of ethane to ethanol over Rh_1-SI/AC catalyst and Rh_1-NI/AC catalyst. 33-34
Supplementary Figure 33. Comparison of rate-determining step ΔE for the reaction pathway of C_2H_6 and O_2 to ethanol on different catalysts. 35

Supplementary Tables

Supplementary Table 1. The specific surface area and pore volume of catalysts...........36

Supplementary Table 2. Curve fit parameters for Rh K-edge EXAFS 37

Supplementary Table 3-5. XPS results 38-40

Supplementary Table 6. Elemental composition for various catalysts 41

Supplementary Table 7. Metal loading amount for the samples based on ICP-OES 42

Supplementary Table 8-10. GC-MS results 43-45

Supplementary Table 11-16. Calculated Reaction energetics of each elementary step for Rh_1-SNI/AC on the reaction path 46-51

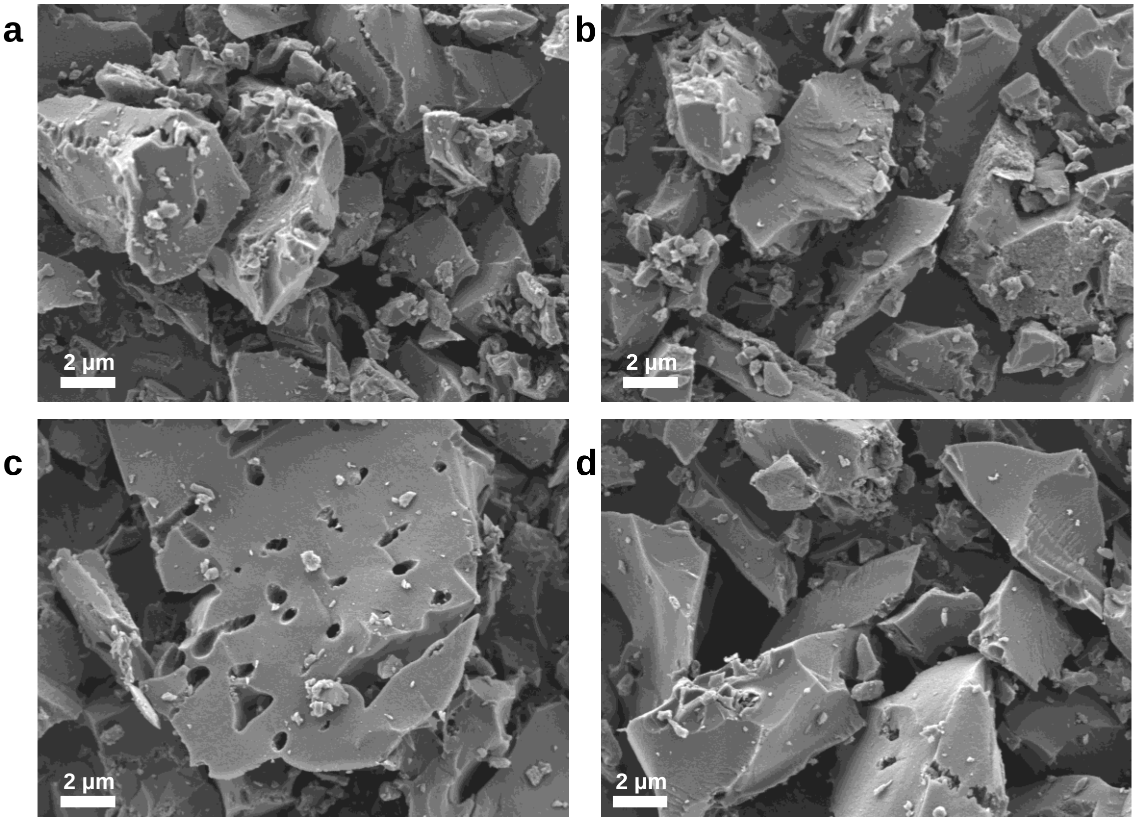

Supplementary Fig. 1 | SEM images of (**a**) fresh Rh_1/AC-NI. (**b**) fresh Rh_1/AC-SI. (**c**) fresh Rh_1/AC-SNI. (**d**) spent Rh_1/AC-SNI.

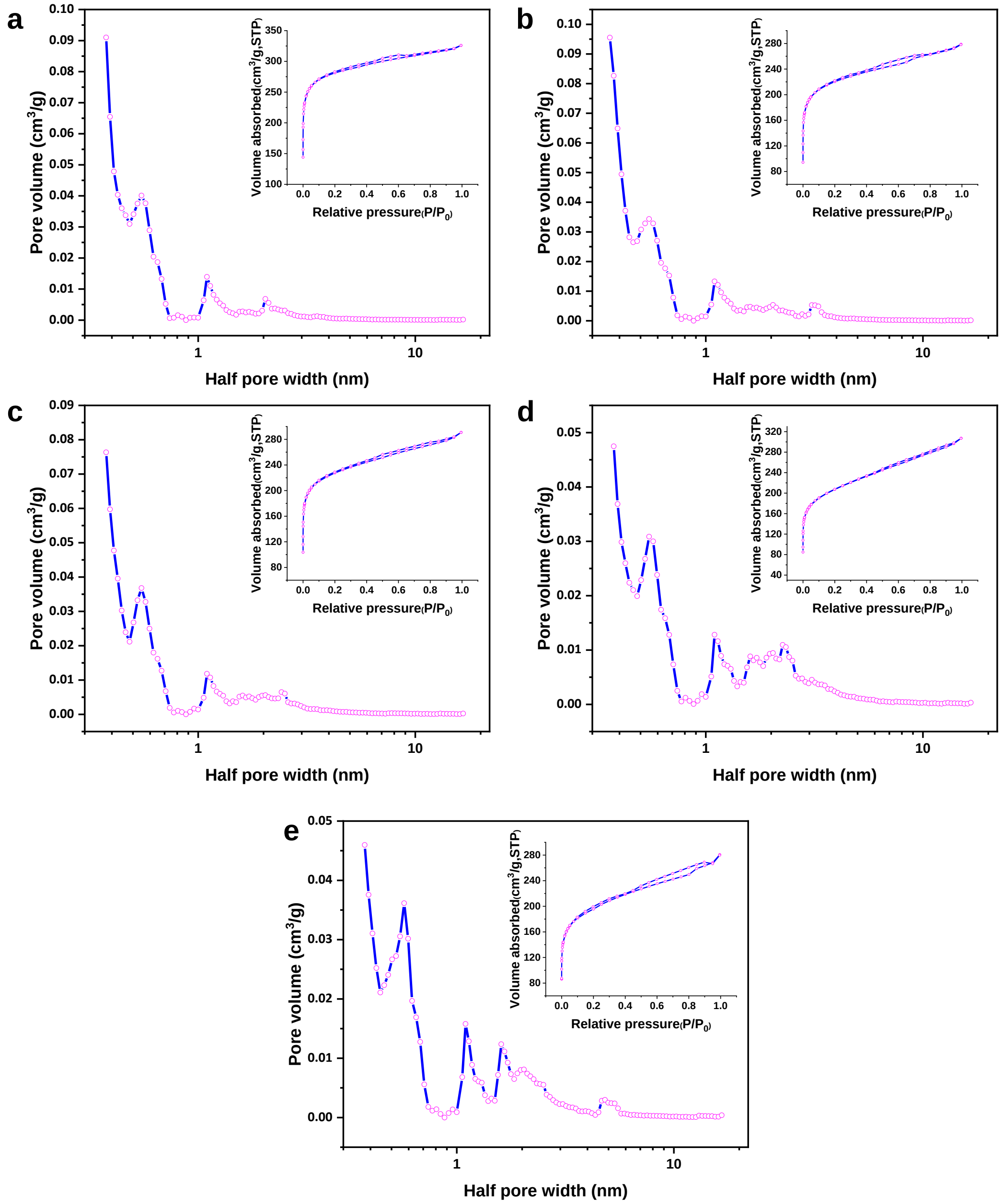

Supplementary Fig. 2 | Pore size distributions and N_2 sorption isotherms (inset) of (a) AC. (**b**) Rh_1/AC-NI. (**c**) Rh_1/AC-SI. (**d**) Rh_1/AC-SNI-fresh. (**e**) spent Rh_1/AC-SNI-spent.

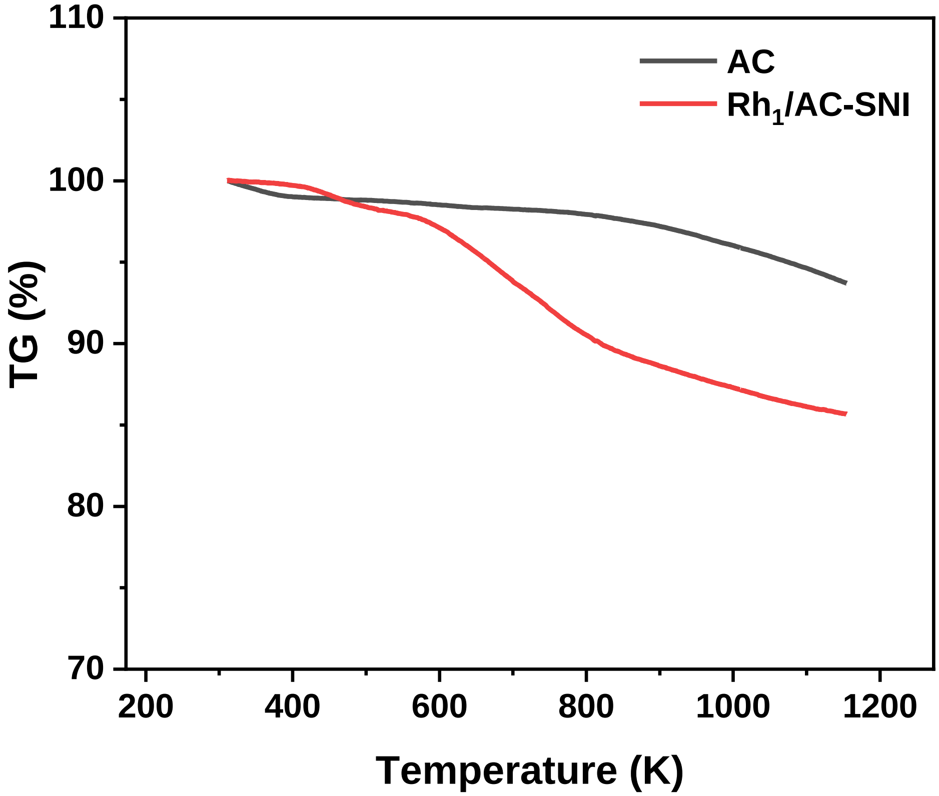

Supplementary Fig. 3 | N$_2$-TG of AC and Rh$_1$/AC-SNI.

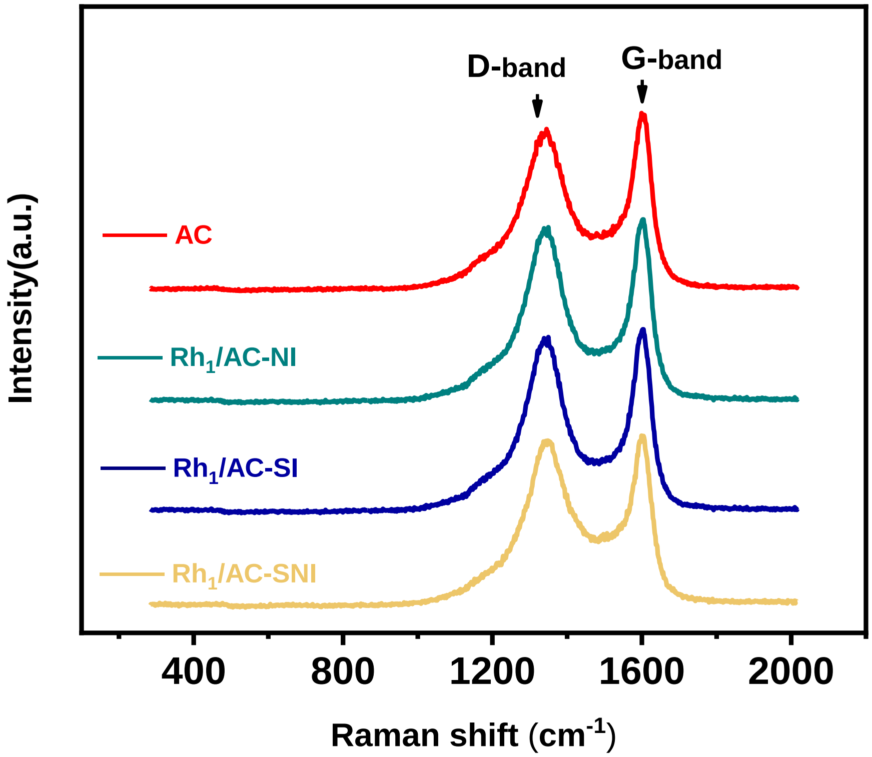

Supplementary Fig. 4 | Raman spectrogram of AC, Rh_1/AC-NI, Rh_1/AC-SI, Rh_1/AC-SNI.

Raman spectroscopy was used to study the degree of defectivity of support and corresponding catalysts. The peak in D band was related to the defects of AC, and the peak in G band denoted the degree of graphitization. The values of I_d/I_g (AC), I_d/I_g (Rh_1/AC-SI), I_d/I_g (Rh_1/AC-NI) and I_d/I_g (Rh_1/AC-SNI) were 1.58, 1.61, 1.62, and 1.67, respectively. It indicated that the defects of the modified AC increase in the order of AC<AC-SI <AC-NI < AC-SNI, which is more favorable to the binding of active metal.

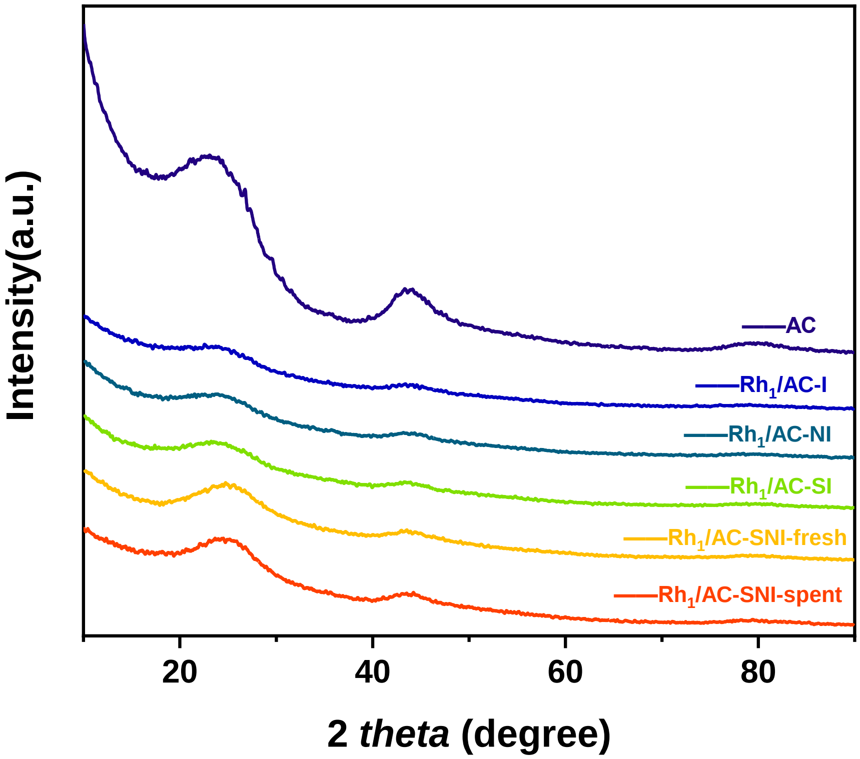

Supplementary Fig. 5 | XRD patterns of AC, Rh/AC-I, Rh_1/AC-NI, Rh_1/AC-SI, fresh Rh_1/AC-SNI and spent Rh_1/AC-SNI.

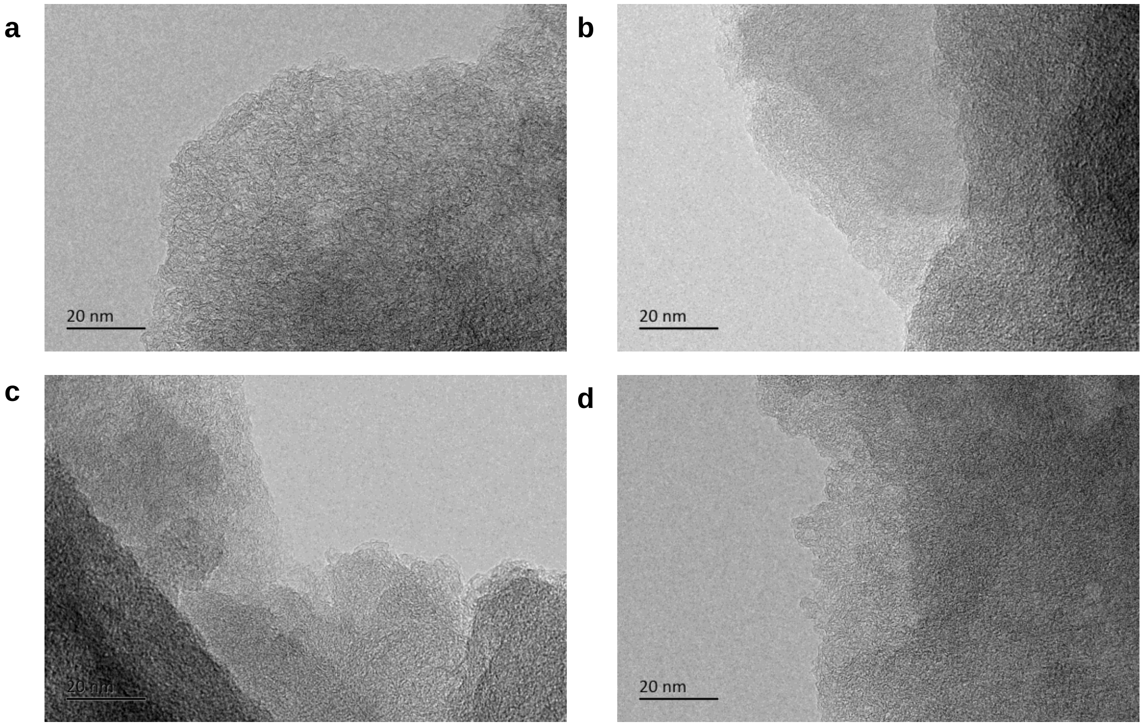

Supplementary Fig. 6 | TEM images of (**a**) Rh_1/AC-NI. (**b**) Rh_1/AC-SI. (**c**) fresh Rh_1/AC-SNI. (**d**) spent Rh_1/AC-SNI.

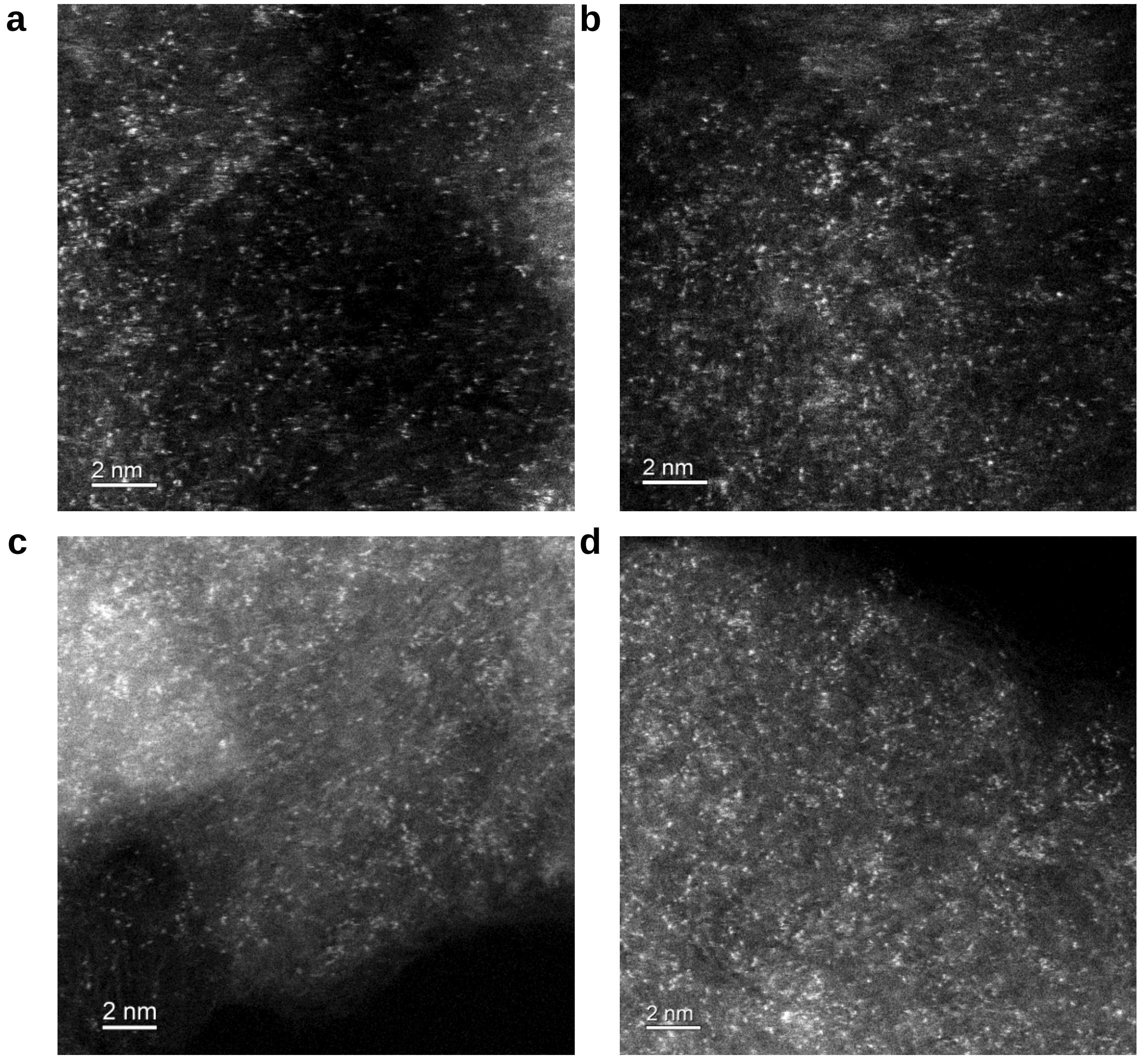

Supplementary Fig. 7 | HAADF-STEM images of (**a**) Rh_1/AC-NI. (**b**) Rh_1/AC-SI. (**c**) fresh Rh_1/AC-SNI. (**d**) spent Rh_1/AC-SNI.

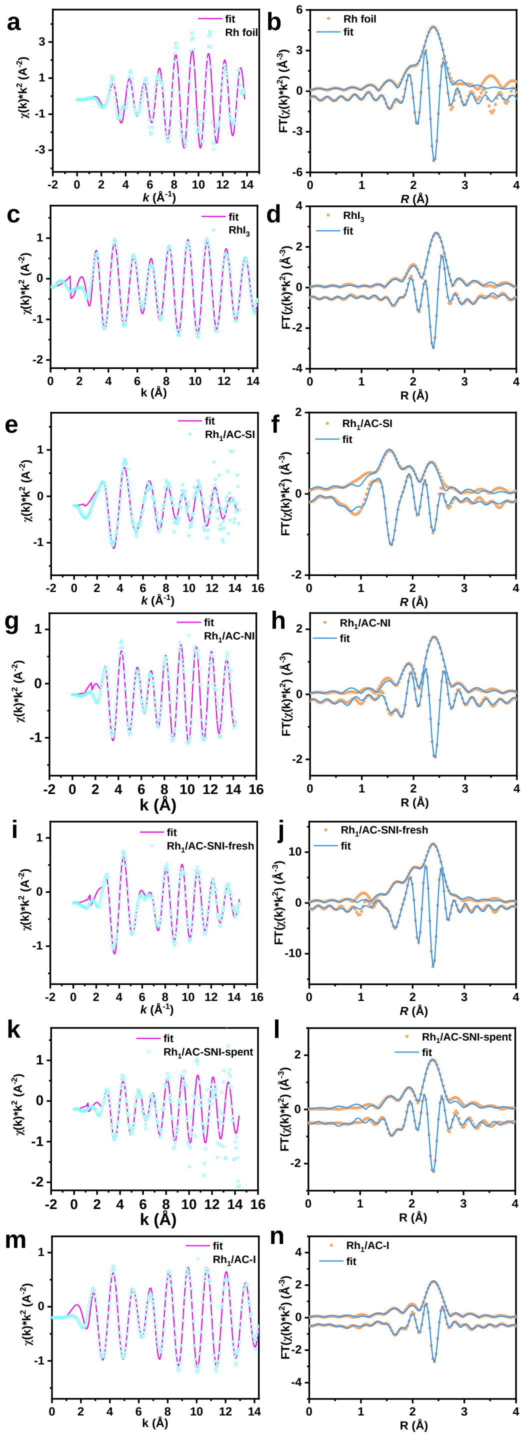

Supplementary Fig. 8 | Fourier-transformed magnitude of Rh K-edge EXAFS spectra in k and R space for (**a**, **b**) Rh foil, (**c**, **d**) RhI_3, (**e**, **f**) Rh_1/AC-SI, (**g**, **h**) Rh_1/AC-NI, (**i**, **j**) fresh Rh_1/AC-SNI, (**k**, **l**) spent Rh_1/AC-SNI and (**m**, **n**) Rh_1/AC-I. Measured and calculated spectra are well matched for all samples.

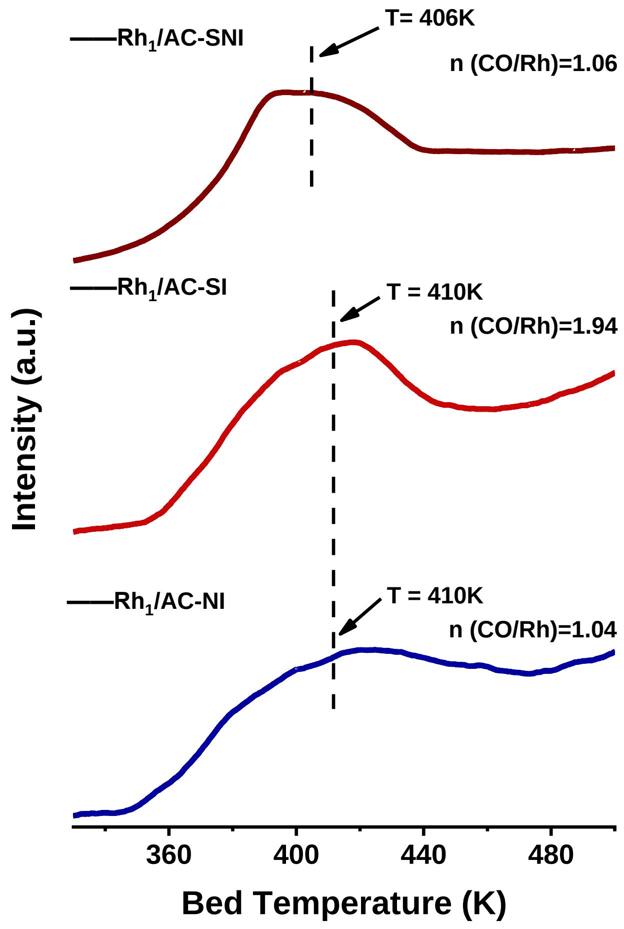

Supplementary Fig. 9 | CO-TPD of Rh_1/AC-SNI, Rh_1/AC-SI and Rh_1/AC-NI.

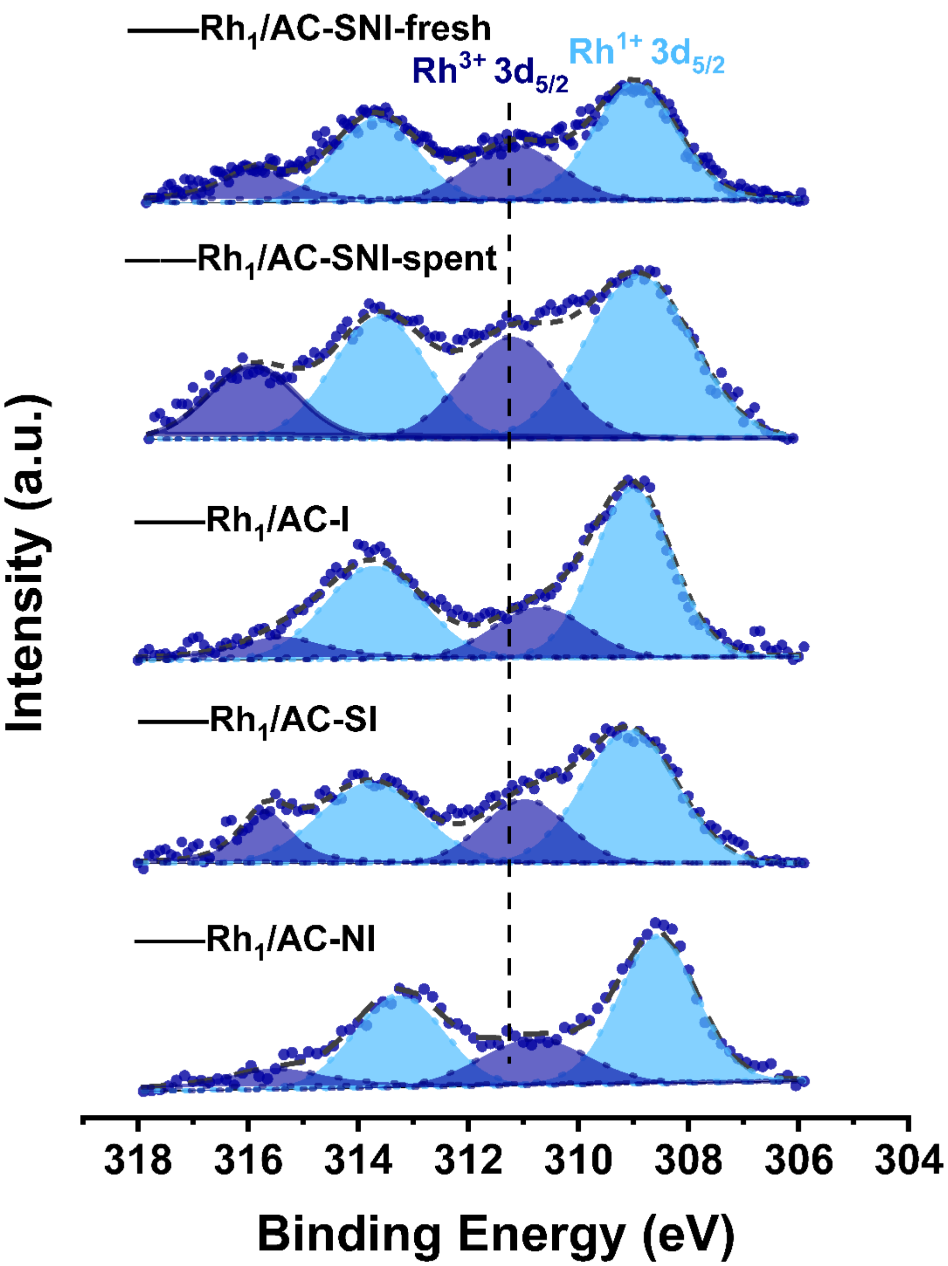

Supplementary Fig. 10 | XPS patterns of Rh 3*d* for Rh_1/AC-I, Rh_1/AC-NI, Rh_1/AC-SI, fresh and spent Rh_1/AC-SNI.

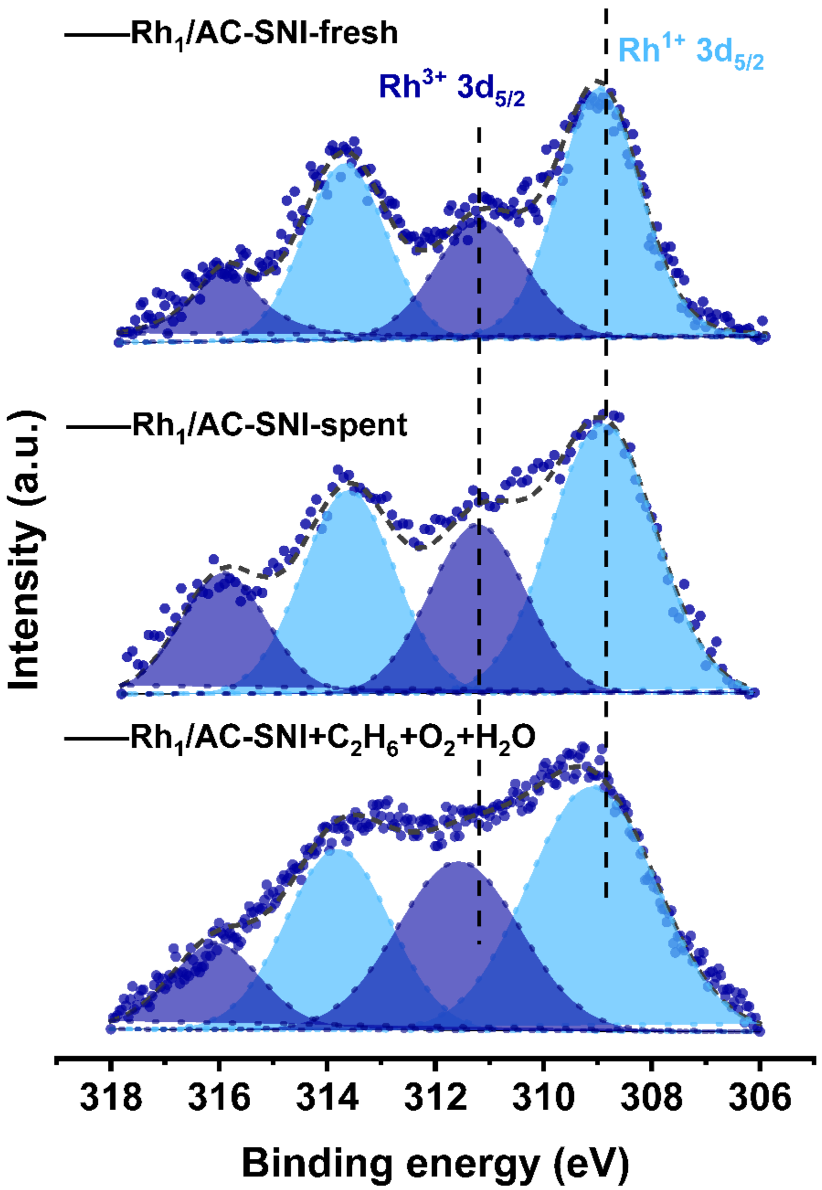

Supplementary Fig. 11 | XPS patterns of Rh 3*d* for fresh Rh_1/AC-SNI, spent Rh_1/AC-SNI and Rh_1/AC-SNI lacking CO atmosphere pretreatment.

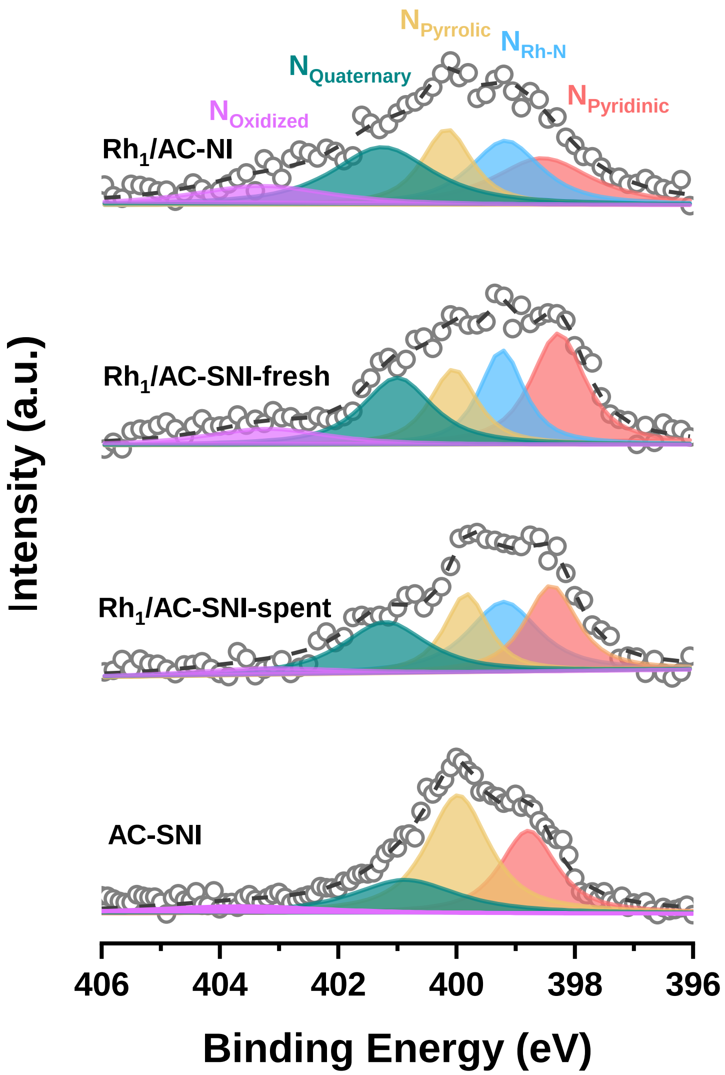

Supplementary Fig. 12 | XPS patterns of N 1*s* for AC-SNI, Rh_1/AC-NI, fresh and spent Rh_1/AC-SNI.

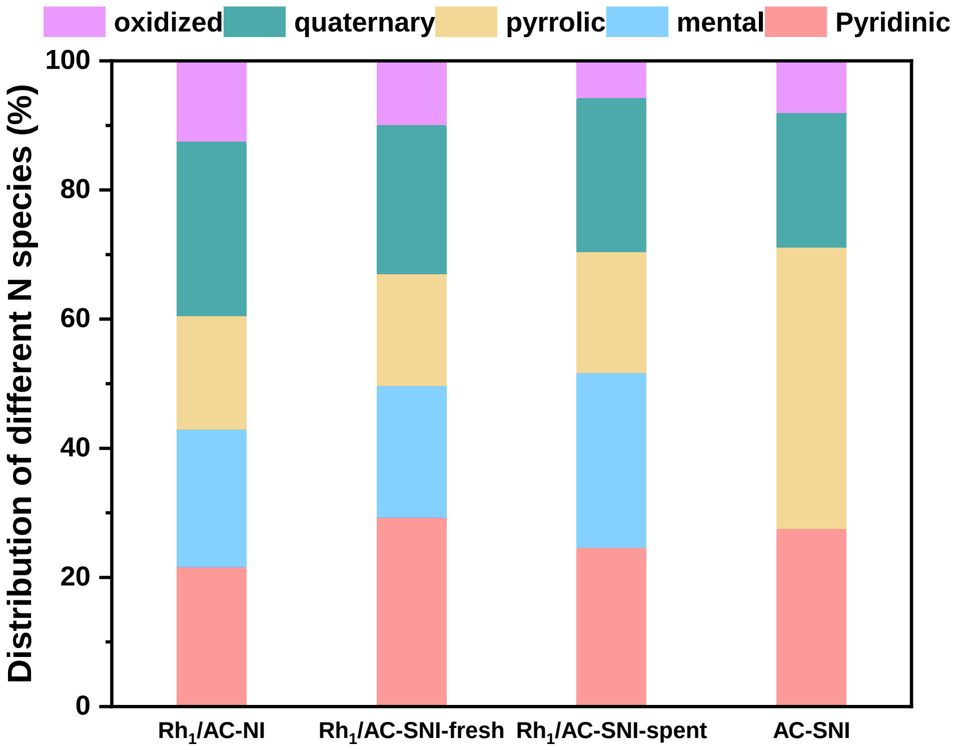

Supplementary Fig. 13 | XPS results of N 1*s* area percent of different N species.

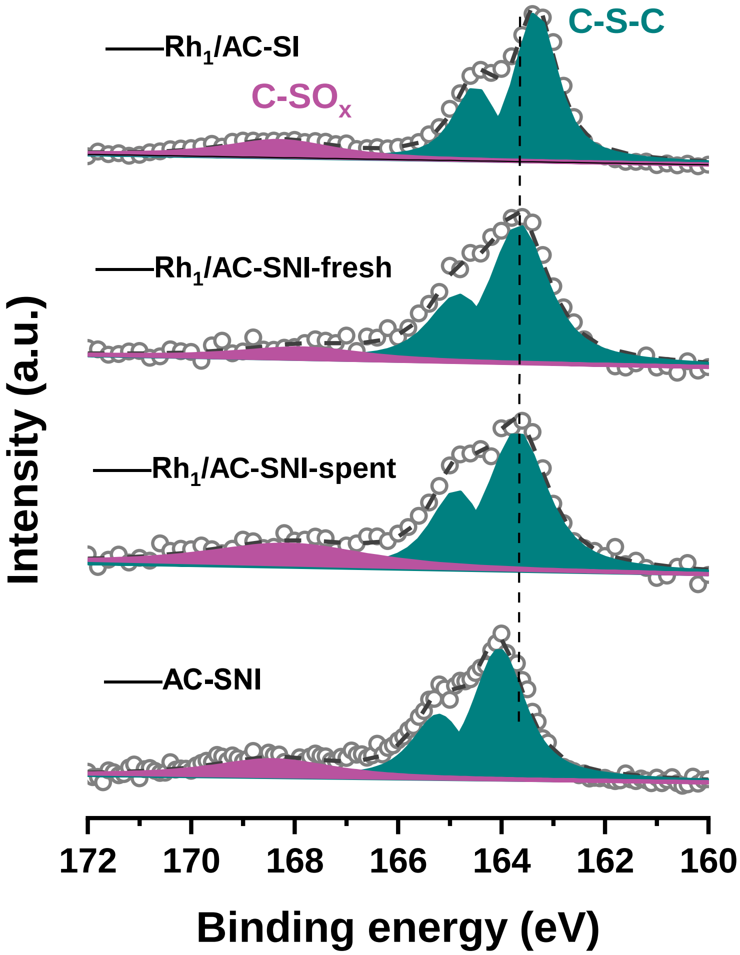

Supplementary Fig. 14 | XPS patterns of S 2*p* for AC-SNI, Rh_1/AC-SI, fresh and spent Rh_1/AC-SNI.

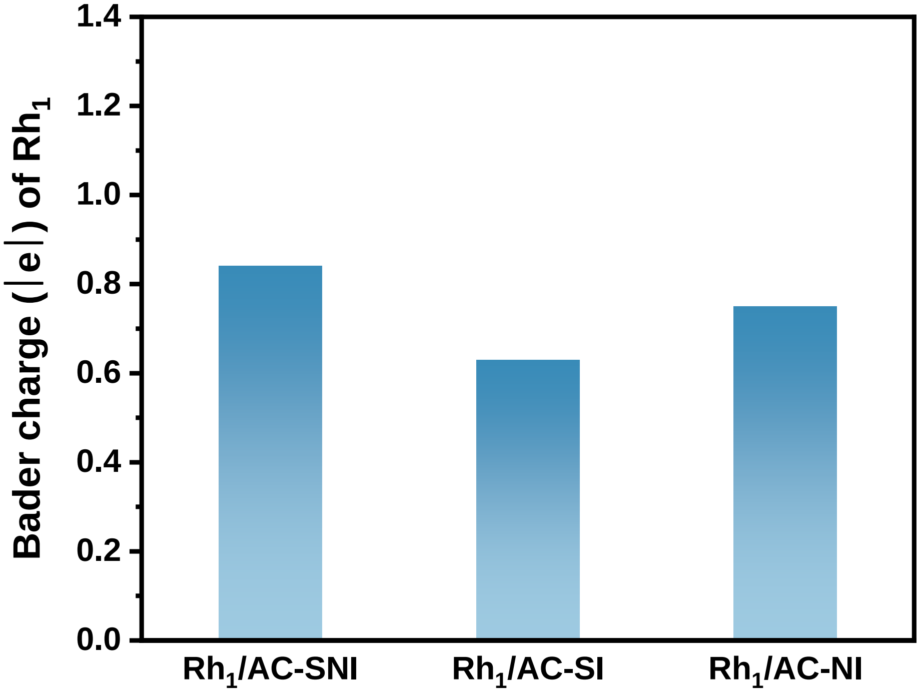

Supplementary Fig. 15 | The Bader charge of Rh_1 on Rh_1/AC-SNI, Rh_1/AC-SI and Rh_1/AC-NI catalysts.

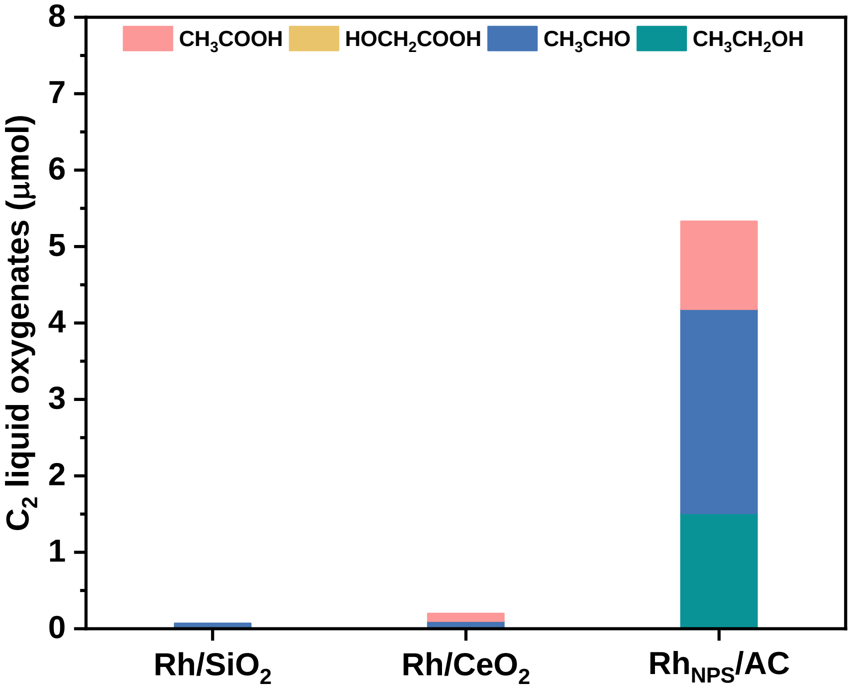

Supplementary Fig. 16 | Activity comparison of different NPs catalysts. Reaction conditions: T = 423 (K), $P_{C_2H_6}$= 3.0 (MPa), P_{CO}=1.0 (MPa) and P_{O_2}= 0.5 (MPa) for 2 (h), m (H_2O) = 10 (g), m (catalyst) = 50 (mg).

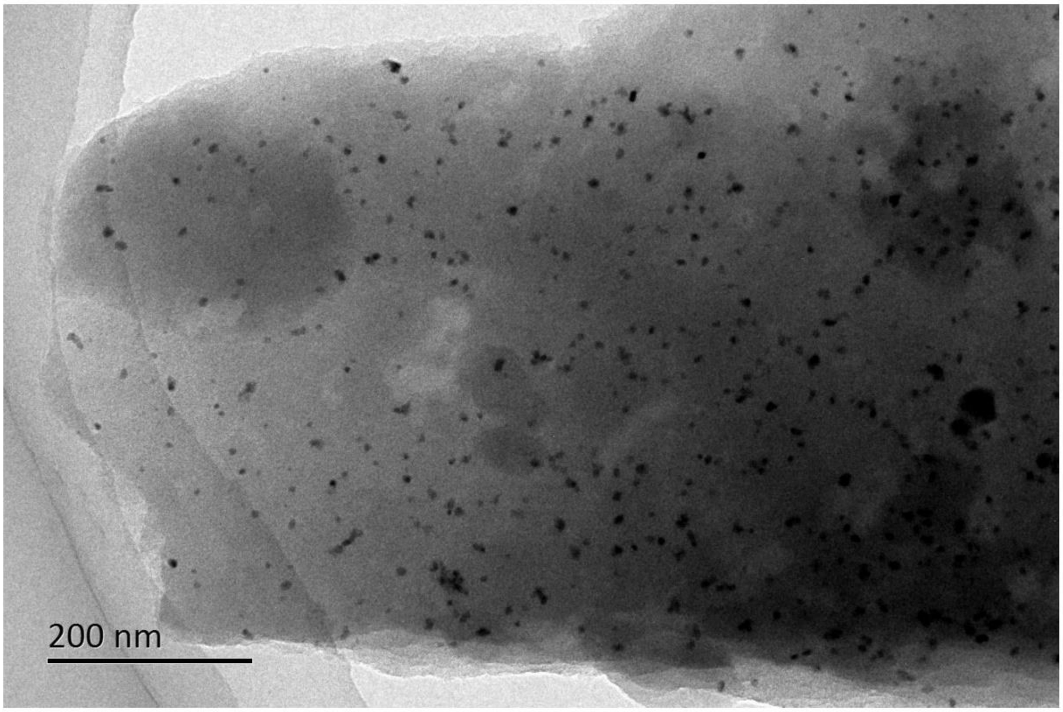

Supplementary Fig. 17 | TEM images of Rh_{NPs}/AC.

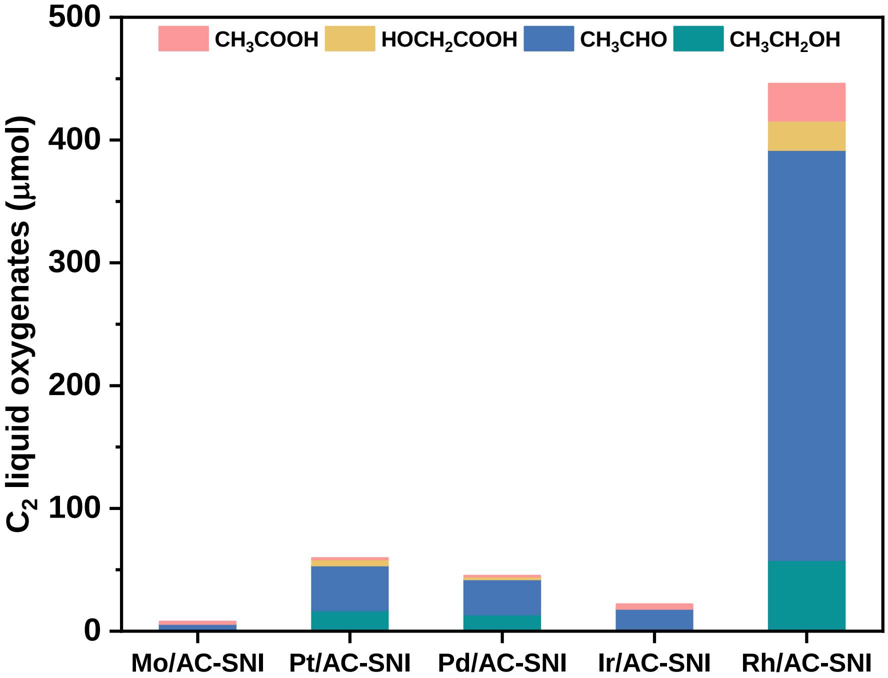

Supplementary Fig. 18 | Activity comparison of different active metal. Reaction conditions: T = 423 (K), $P_{C_2H_6}$= 3.0 (MPa), P_{CO}=1.0 (MPa) and P_{O_2}= 0.5 (MPa) for 2 (h), m (H_2O) = 10 (g), m (catalyst) = 50 (mg).

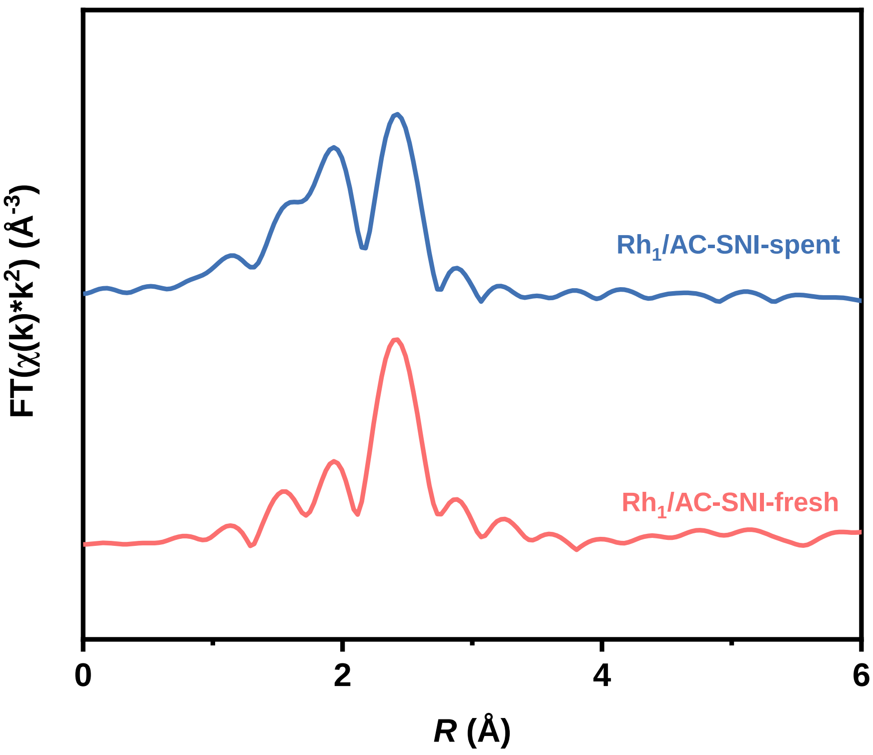

Supplementary Fig. 19 | The experimental curve of k^1 -weight EXAFS spectra in r-space of fresh Rh_1/AC-SNI and spent Rh_1/AC-SNI.

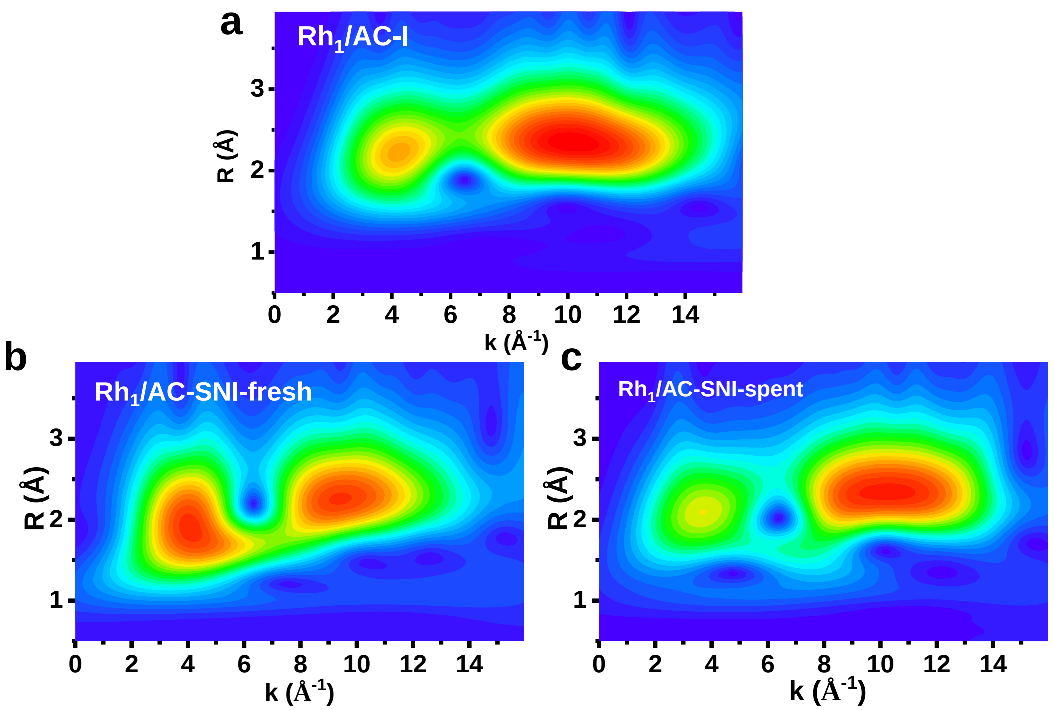

Supplementary Fig. 20 | The wavelet transform contour plots of k^1 -weighted χ(k) EXAFS signals of (**a**) Rh_1/AC-I, (**b**) fresh Rh_1/AC-SNI and (**c**) spent Rh_1/AC-SNI.

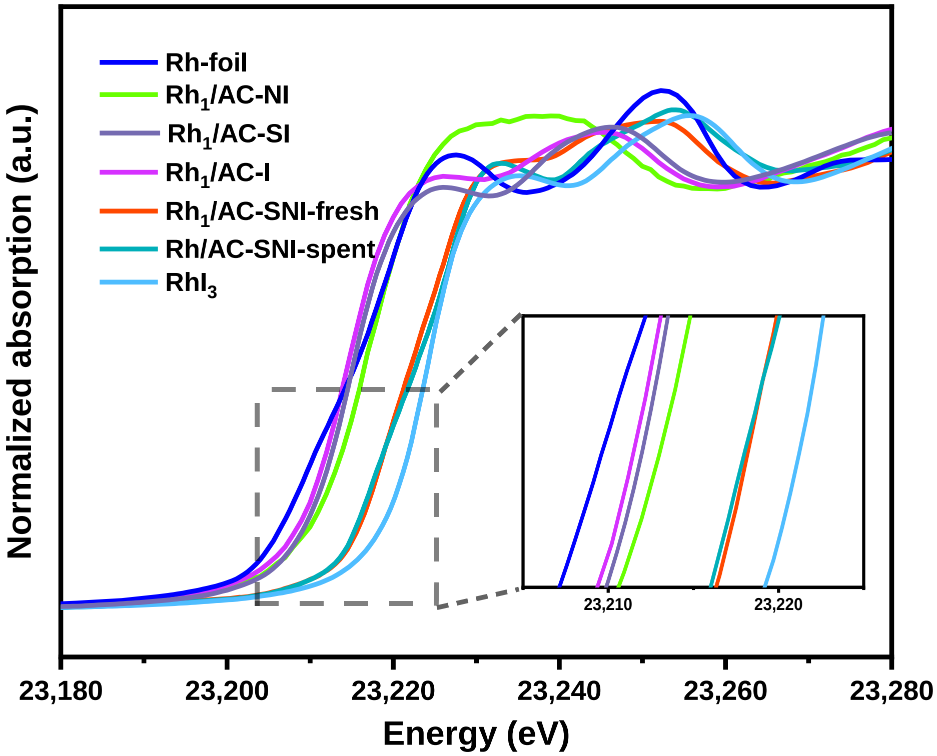

Supplementary Fig. 21 | XANES spectra for Rh foil, RhI_3, fresh Rh_1/AC-SNI, spent Rh_1/AC-SNI, Rh_1/AC-NI, Rh_1/AC-SI and Rh_1/AC-I.

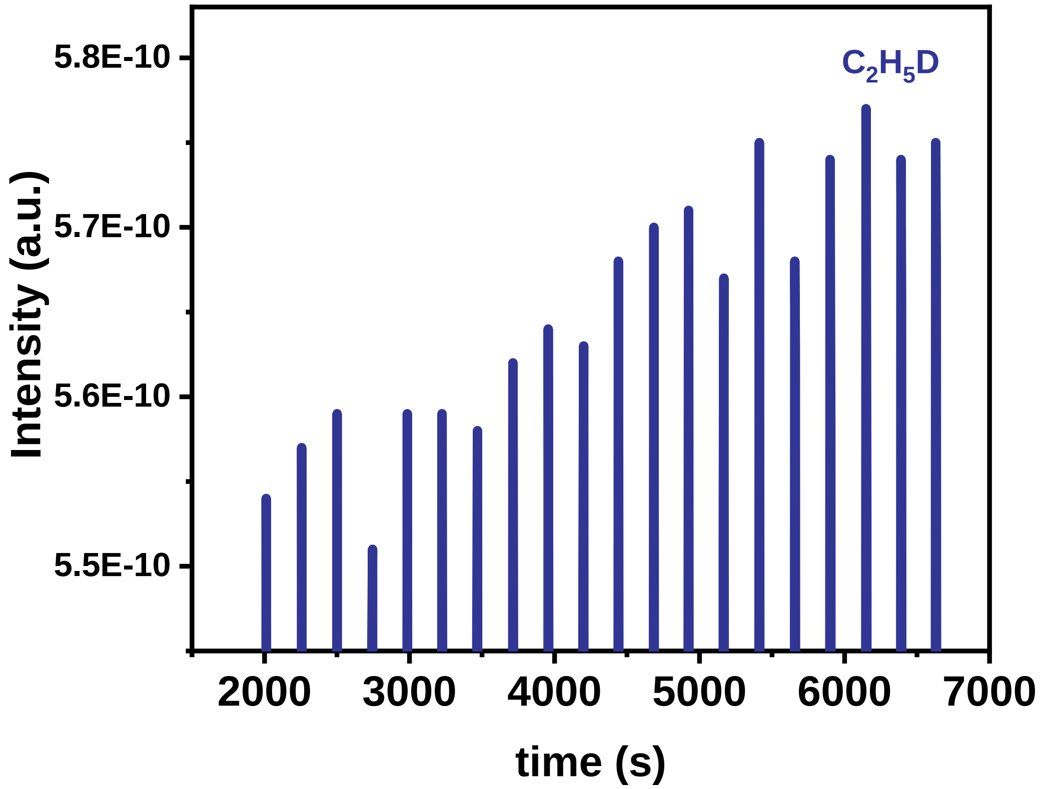

Supplementary Fig. 22 | C_2H_6-D_2-TPD-MS experiment**.**

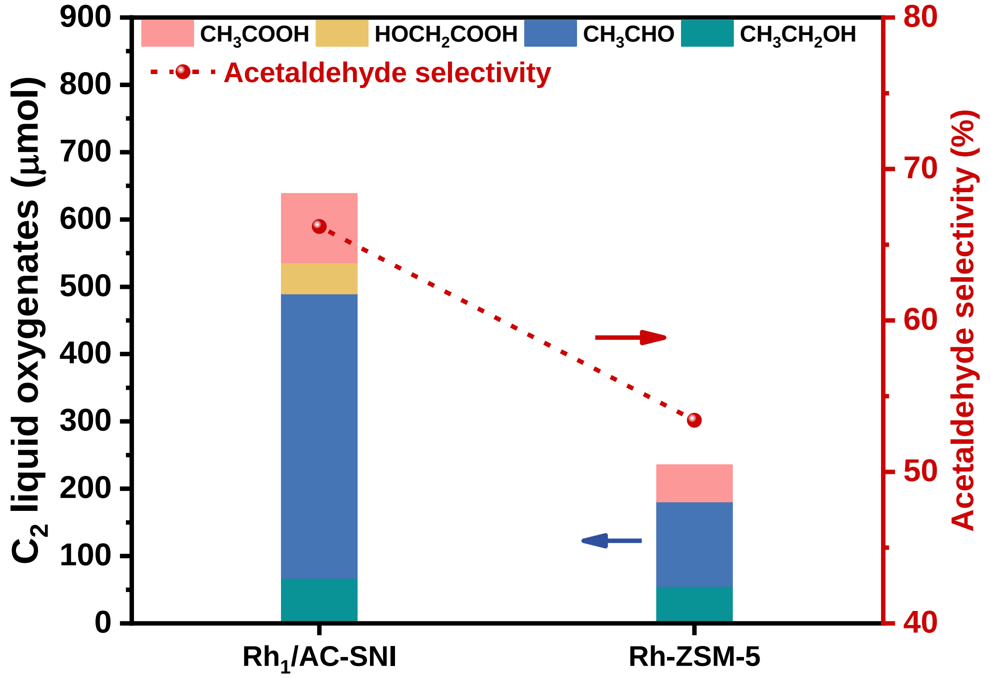

Supplementary Fig. 23 | Activity comparison of Rh1/AC-SNI and Rh-ZSM-5. Reaction conditions: T = 423 (K), $P_{C_2H_6}$= 3.0 (MPa), P_{CO}=1.0 (MPa) and P_{O_2}= 0.5 (MPa) for 2 (h), m (H_2O) = 10 (g), m (catalyst) = 50 (mg).

Rh-ZSM-5

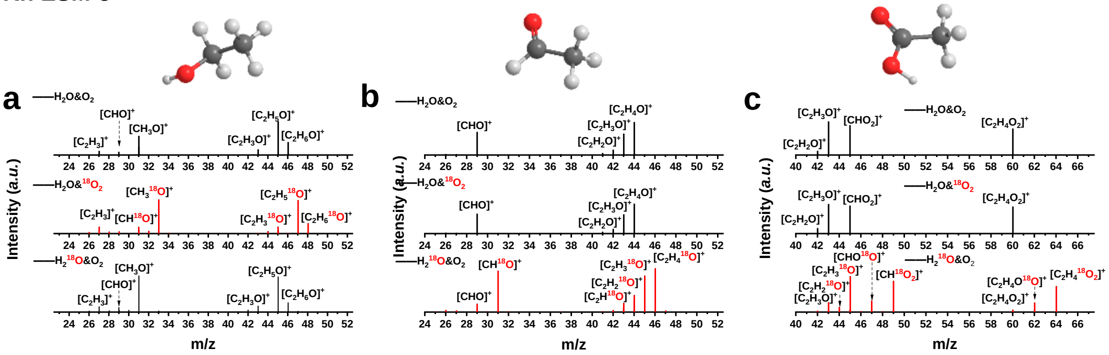

Supplementary Fig. 24 | GC-MS spectra of (**a**) ethanol (**b**) acetaldehyde (**c**) acetic acid, respectively with H_2O/ $^{18}O_2$/ $H_2{}^{18}O$ isotope labeling experiment on Rh-ZSM-5 catalyst.

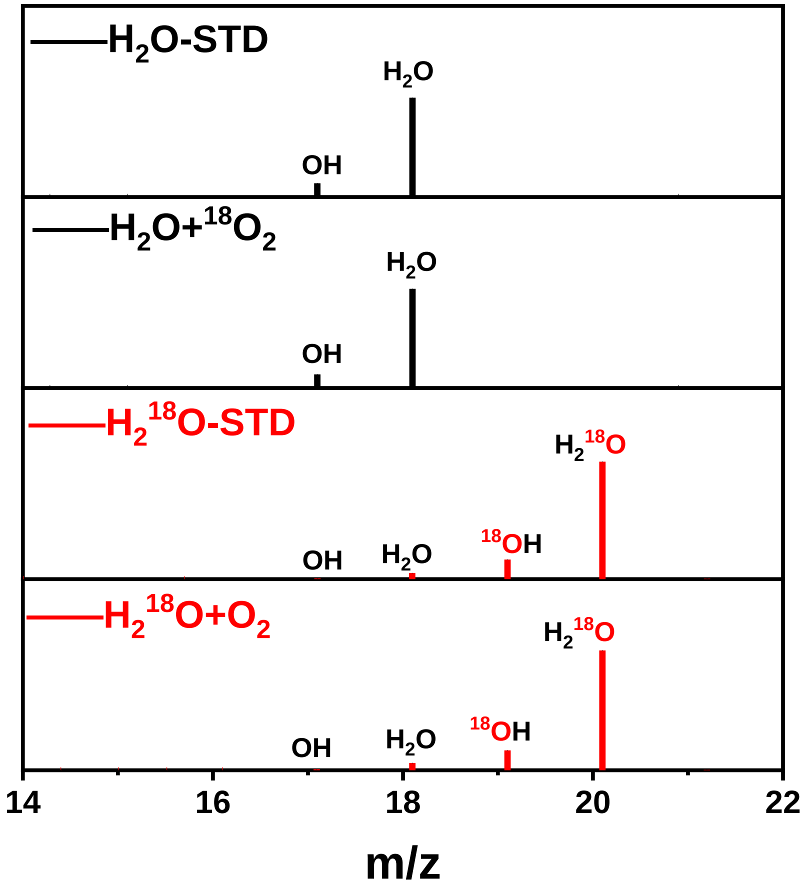

Supplementary Fig. 25 | GC-MS spectra of H_2O**.**

To further verify whether the O exchange between H_2O and O_2 occurs under the reaction conditions, we conducted two controlled experiments. H_2O and 1 bar $^{18}O_2$ were mixed at 423K with the total pressure of 45 bar (remaining N_2) over 50mg Rh_1/AC-SNI catalyst. $H_2{}^{18}O$ and 5 bar O_2 were mixed at 423K with the total pressure of 45 bar (remaining N_2) over 50mg Rh_1/AC-SNI catalyst. H_2O-STD and $H_2{}^{18}O$-STD were the standard samples of H_2O and $H_2{}^{18}O$, respectively. The reaction results indicated that almost negligible O was exchanged during the reaction.

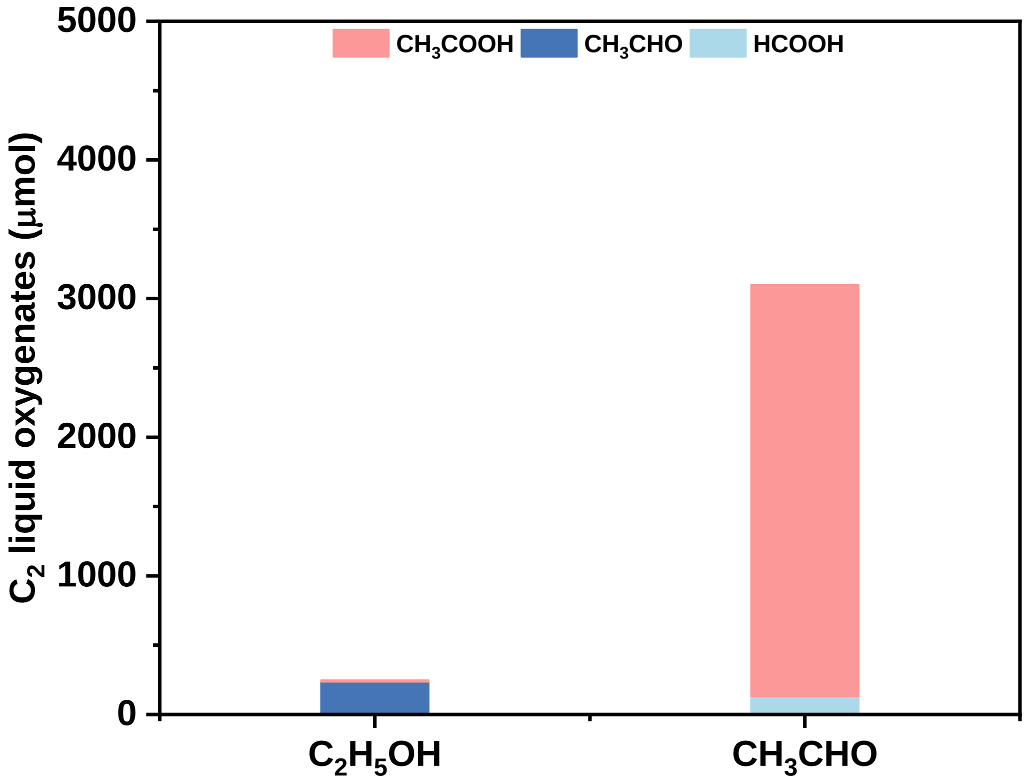

Supplementary Fig. 26 | Comparison of products using ethanol and acetaldehyde as substrates.

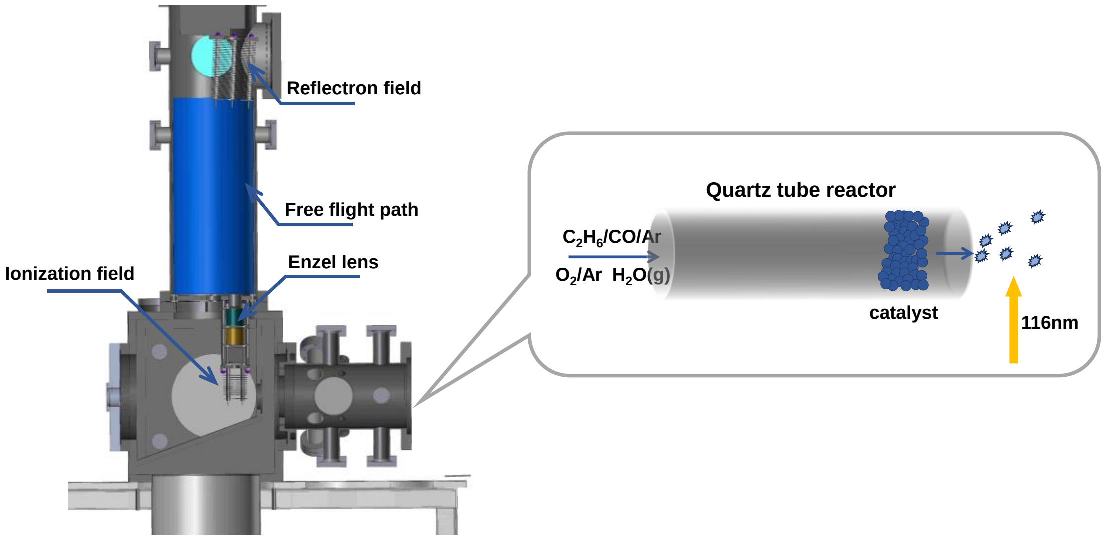

Supplementary Fig. 27 | Schematic diagram of the experimental setup and quartz tube reactor.

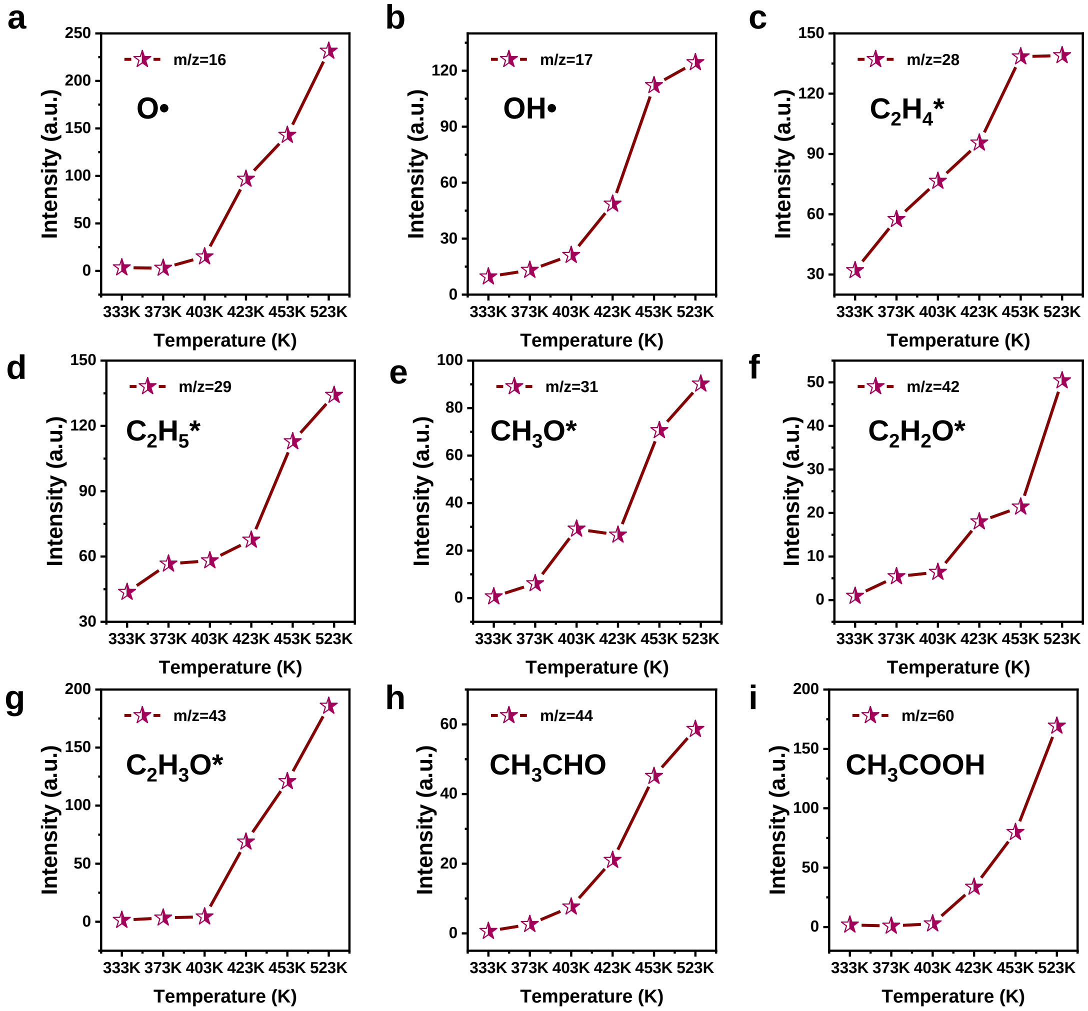

Supplementary Fig. 28 | The signal of (**a**) O•, (**b**) OH•, (**c**) C_2H_4*, (**d**) C_2H_5*, (**e**) CH_3O*, (**f**) C_2H_2O*, (**g**) C_2H_3O* (**h**) CH_3CHO, (**i**) CH_3COOH change with temperature increase of in situ free-electron laser time of flight mass spectrometry.

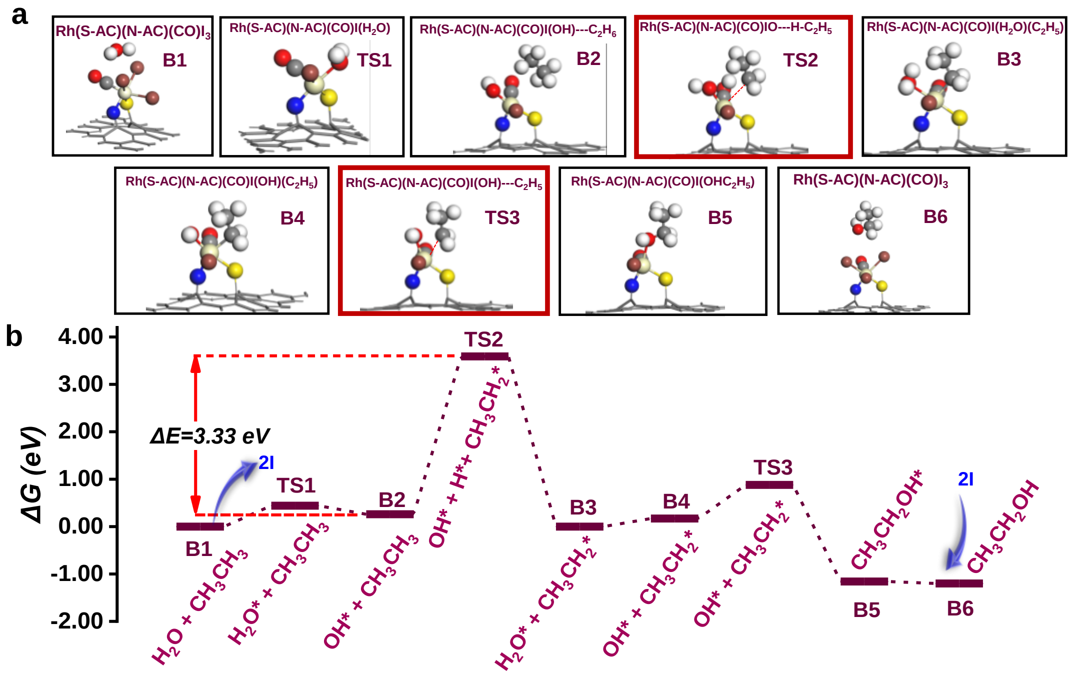

Supplementary Fig. 29 | DFT calculations. (**a**) Structures of the key intermediates involved in the hypothesized reaction pathway of C_2H_6 and H_2O to ethanol on Rh_1/AC-SNI catalyst. (**b**) The free energy (ΔG) diagrams of the hypothesized reaction pathway of H_2O participating in the production of ethanol. The states B1-B6 represent different basic states in the reaction pathway, and TS represents the transition state. Colors in the picture: the white balls are H; the gray balls are C; the red balls are O; the blue balls are N; the yellow balls are S; the brown balls are I; the beige balls are Rh.

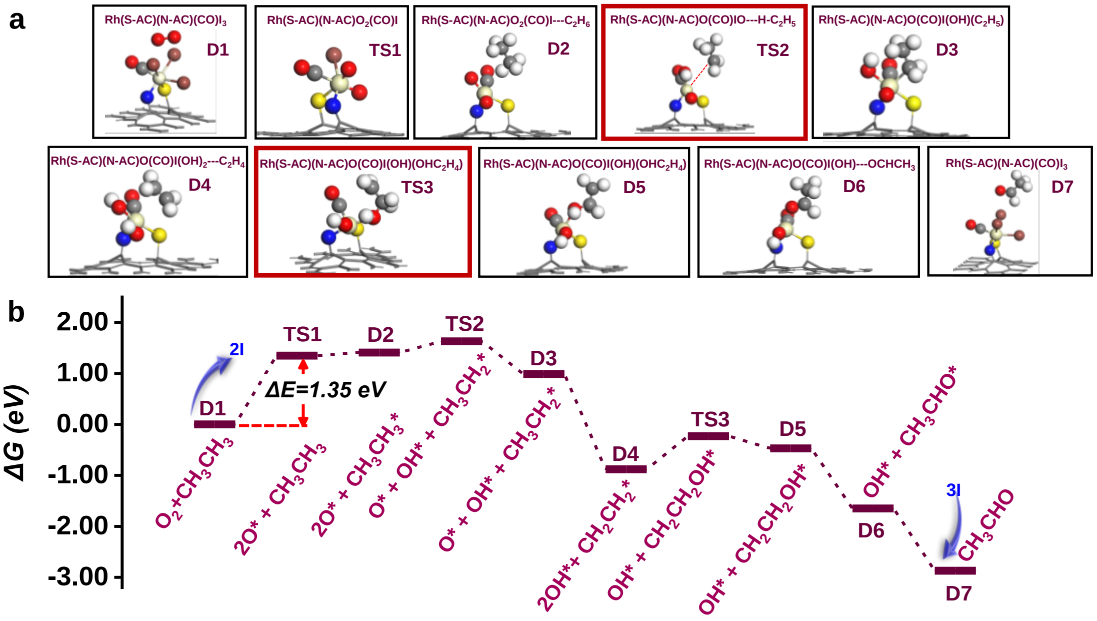

Supplementary Fig. 30 | DFT calculations. (**a**) Structures of the key intermediates involved in the hypothesized reaction pathway of C_2H_6 and O_2 to acetaldehyde on Rh_1/AC-SNI catalyst. (**b**) The free energy (ΔG) diagrams of the hypothesized reaction pathway of O_2 participating in the production of acetaldehyde. The states D1-D7 represent different basic states in the reaction pathway, and TS represents the transition state. Colors in the picture: the white balls are H; the gray balls are C; the red balls are O; the blue balls are N; the yellow balls are S; the brown balls are I; the beige balls are Rh.

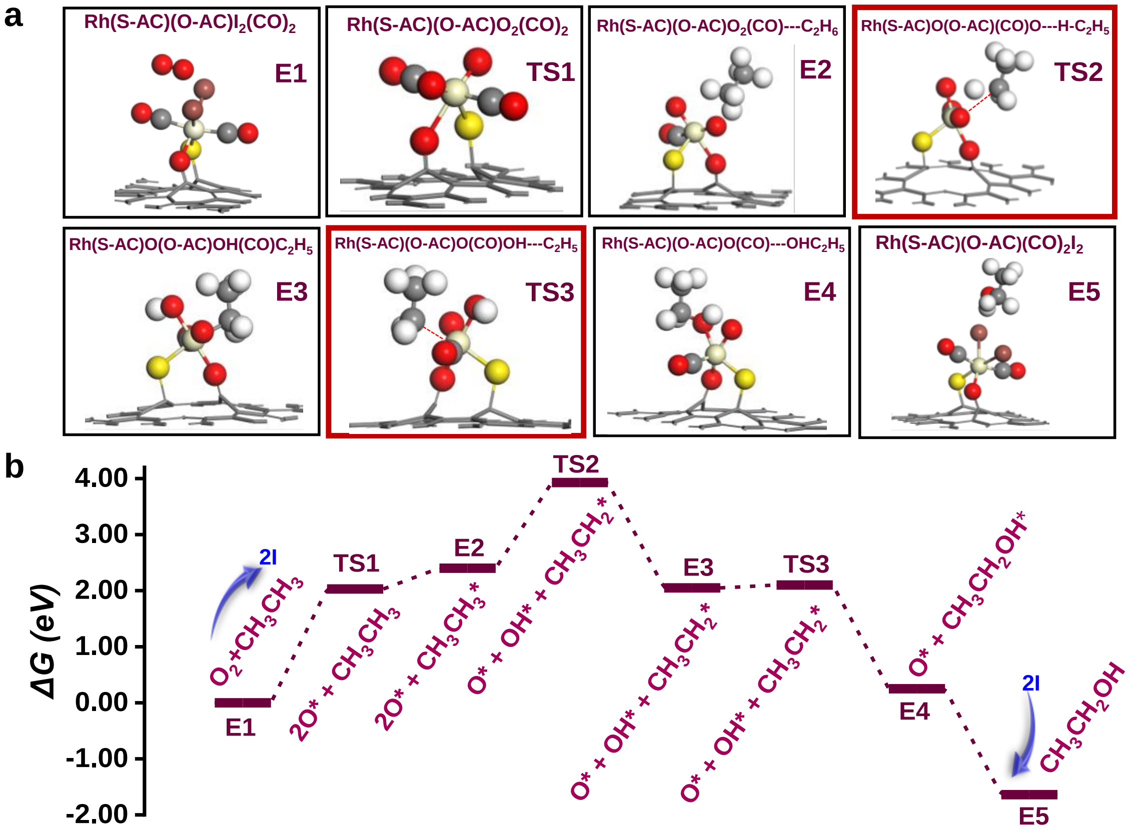

Supplementary Fig. 31 | DFT calculations. (**a**) Structures of the key intermediates involved in the hypothesized reaction pathway of C_2H_6 and O_2 to ethanol on Rh_1/AC-SI catalyst. (**b**) The free energy (ΔG) diagrams of the hypothesized reaction pathway of O_2 participating in the production of acetaldehyde. The states E1-E5 represent different basic states in the reaction pathway, and TS represents the transition state. Colors in the picture: the white balls are H; the gray balls are C; the red balls are O; the blue balls are N; the yellow balls are S; the brown balls are I; the beige balls are Rh.

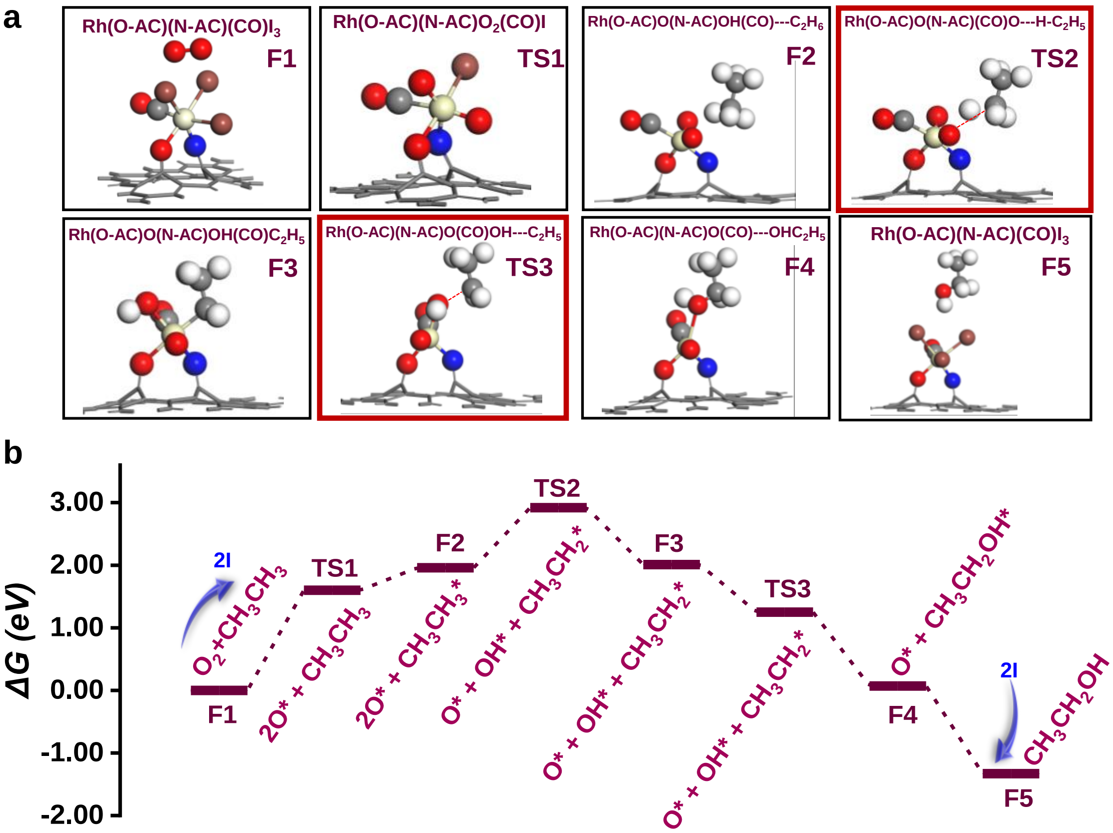

Supplementary Fig. 32 | DFT calculations. (**a**) Structures of the key intermediates involved in the hypothesized reaction pathway of C_2H_6 and O_2 to ethanol on Rh_1/AC-NI catalyst. (**b**) The free energy (ΔG) diagrams of the hypothesized reaction pathway of O_2 participating in the production of acetaldehyde. The states F1-F5 represent different basic states in the reaction pathway, and TS represents the transition state. Colors in the picture: the white balls are H; the gray balls are C; the red balls are O; the blue balls are N; the yellow balls are S; the brown balls are I; the beige balls are Rh.

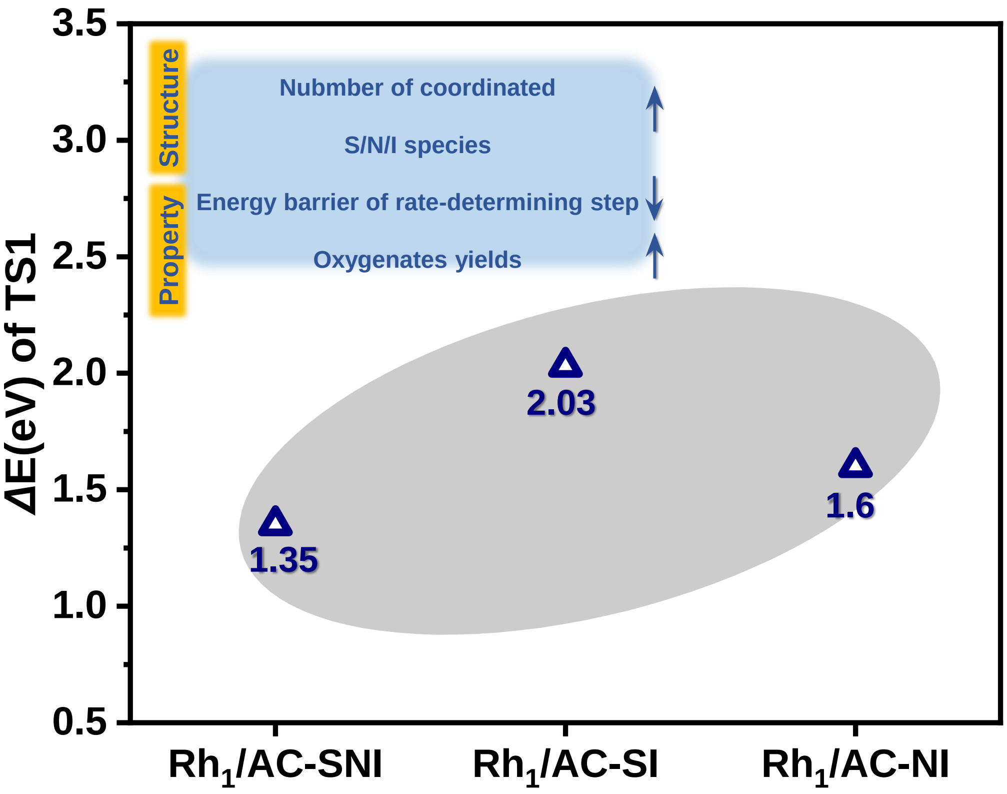

Supplementary Fig. 33 | Comparison of rate-determining step ΔE for the reaction pathway of C_2H_6 and O_2 to ethanol on different catalysts.

2.Supplementary Tables

Supplementary Table 1 | The specific surface area and pore volume of AC, Rh_1/AC-NI, Rh_1/AC-SI, fresh Rh_1/AC-SNI and spent Rh_1/AC-SNI

Catalysts	specific surface areas (m^2/g)	pore volume (cm^3/g)
AC	868	0.461
Rh_1/AC-NI	693	0.396
Rh_1/AC-SI	716	0.432
Rh_1/AC-SNI-fresh	673	0.417
Rh_1/AC-SNI-spent	634	0.396

Supplementary Table 2 | Curve fit parameters of Rh K-edge EXAFS for various catalysts.

Sample	Path	CN	R (Å)	σ^2 ($\times10^{-3}$ Å^2)	ΔE_0 (eV)	R (%)
Rh foil [a]	Rh-Rh$_1$	12	2.68±0.00	3.2±0.4	-6.8±0.6	1
	Rh-C/N	1.4±0.2	2.07±0.04	3	4.0±8.6	
Fresh Rh$_1$/AC-SNI [b]	Rh-S	0.7±0.2	2.35±0.03	3	-0.7±4.7	0.2
	Rh-I	2.6±0.2	2.66±0.00	3.5±0.5	7.7±0.5	
	Rh-C/N	2.1±0.6	2.11±0.06	5	5.7±0.6	
Spent Rh$_1$/AC-SNI [c]	Rh-S	1.1±0.5	2.36±0.04	1.2	9.8±0.9	0.8
	Rh-I	2.5±0.2	2.65±0.01	3	4.6±1.2	
	Rh-C/O	3.0±0.6	2.03±0.03	3	3.3±5.2	
Fresh Rh$_1$/AC-SI [d]	Rh-S	0.9±0.1	2.36±0.05	3	2.6±7.6	1.6
	Rh-I	2.1±0.3	2.62±0.03	3	-1.1±5.4	
Fresh Rh$_1$/AC-NI [e]	Rh-O/N/C	2.9±0.4	2.24±0.08	13.3±1.4	9.7±5.1	0.4
	Rh-I	3.1±0.5	2.65±0.01	3.3±0.1	5.0±0.9	
Rh$_1$/AC-I [f]	Rh-C/O	1.9±0.4	2.07±0.04	2.7±0.9	-2.39±0.99	0.3
	Rh-I	3.7±0.3	2.66±0.00	3.9±0.3	4.48±0.48	
RhI_3 [g]	Rh-I	6	2.65±0.00	4.1±0.3	7.1±0.3	0.2

N, coordination number; R, distance between absorber and backscatter atoms; σ^2, Debye-Waller factor to account for both thermal and structural disorders; ΔE_0, inner potential correction; R factor (%) indicates the goodness of the fit. S_0^2 was fixed to 0.85 as determined from Rh foil fitting.

[a] Fitting range: $2.5 \leq k$ (/Å) ≤ 11.8 and $1.0 \leq R$ (/Å) ≤ 3.2.

[b] Fitting range: $3.1 \leq k$ (/Å) ≤ 13.8 and $1.2 \leq R$ (/Å) ≤ 3.0.

[c] Fitting range: $2.4 \leq k$ (/Å) ≤ 12.5 and $1.0 \leq R$ (/Å) ≤ 2.7.

[d] Fitting range: $2.8 \leq k$ (/Å) ≤ 12.6 and $1.0 \leq R$ (/Å) ≤ 2.9.

[f] Fitting range: $3.0 \leq k$ (/Å) ≤ 13.8 and $1.1 \leq R$ (/Å) ≤ 3.0.

[g] Fitting range: $2.4 \leq k$ (/Å) ≤ 11.8 and $1.3 \leq R$ (/Å) ≤ 3.0.

Supplementary Table 3 | XPS results of Rh 3d for Rh/AC-I, Rh_1/AC-NI, Rh_1/AC-SI, fresh Rh_1/AC-SNI fresh Rh_1/AC-SNI, spent Rh_1/AC-SNI and Rh_1/AC-SNI+C_2H_6+H_2O+O_2.

Catalyst	Rh^{1+}		Rh^{3+}	
	B.E. (eV)	Area (%)	B.E. (eV)	Area (%)
Rh_1/AC-I	309.01	74.21%	310.71	25.79%
Rh_1/AC-SI	308.56	71.08%	310.80	28.92%
Rh_1/AC-NI	309.07	69.65%	310.97	30.35%
Rh_1/AC-SNI-fresh	308.97	65.76%	311.26	34.24%
Rh_1/AC-SNI-spent	308.92	63.65%	311.32	36.35%
Rh_1/AC-SNI +C_2H_6+H_2O+O_2	309.10	60.16%	311.57	39.84%

Supplementary Table 4 | XPS results of N 1s for Rh/AC-NI, fresh Rh_1/AC-SNI, spent Rh_1/AC-SNI and AC-SNI.

N species	Rh_1/AC-NI		Rh_1/AC-SNI-fresh		Rh_1/AC-SNI-spent		AC-SNI	
	B.E.(eV)	Area(%)	B.E.(eV)	Area(%)	B.E.(eV)	Area(%)	B.E.(eV)	Area(%)
Pyridinic	398.54	21.55%	398.28	29.23%	398.4	24.55%	398.78	27.5%
Metal	399.17	21.33%	399.23	20.44%	399.2	27.08%	/	/
Pyrrolic	400.16	17.58%	400.05	17.35%	399.83	18.76%	399.97	43.57%
Quaternary	401.25	27.01%	401.00	23.02%	401.21	23.82%	400.86	20.83%
Oxidized	403.3	12.53%	403.2	9.96%	403.2	5.79%	403.66	8.10%

Supplementary Table 5 | XPS results of S 2p for Rh_1/AC-SI, fresh Rh_1/AC-SNI, spent Rh_1/AC-SNI and AC-SNI.

Catalyst	C-S-C		$C\text{-}SO_x$	
	B.E. (eV)	Area (%)	B.E. (eV)	Area (%)
Rh_1/AC-SI	163.34	83.60%	168.3	16.40%
Rh_1/AC-SNI-fresh	163.67	89.35%	167.84	10.65%
Rh_1/AC-SNI-spent	163.7	76.62%	168.14	23.38%
AC-SNI	164.05	82.34%	168.42	17.66%

Supplementary Table 6 | Elemental composition for various catalysts.

Entry	Catalysts	Mental content (wt.%)	N content (wt.%)	S content (wt.%)
1	Rh_1/AC-I	0.95	-	-
2	Rh_1/AC-NI	0.99	4.57	-
3	Rh_1/AC-SI	0.97	-	2.03
4	Rh_1/AC-SN	0.94	1.46	0.92
5	Rh_1/AC-SNI-fresh	0.93	1.47	0.91
6	Rh_1/AC-SNI-spent	0.91	1.37	0.83

Supplementary Table 7 | Metal loading amount for the samples based on ICP-OES.

Entry	Catalysts	Mental content (wt.%)
1	Rh/SiO_2	0.87
2	Rh/CeO_2	0.98
3	Mo/AC-SNI	0.28
4	Pd/AC-SNI	0.17
5	Pt/AC-SNI	0.97
6	Ir/AC-SNI	0.47
7	Rh_{Nps}/AC	0.99
8	Rh_1/AC-SNI	0.93

Supplementary Table 8 | GC-MS results of C_2H_5OH for various isotopically labeled reactants.

isotope	C_2H_5OH	m/z
H_2O+O_2	$[C_2H_3]^+$	27.0229
	$[CH_3O]^+$	31.0179
	$[C_2H_5O]^+$	45.0334
D_2O+O_2	$[CH_3O]^+$	31.0179
	$[CH_2DO]^+$	32.0242
	$[C_2H_5O]^+$	45.0335
	$[C_2H_4DO]^+$	46.0402
$H_2O+^{18}O_2$	$[CH_3O]^+$	31.0177
	$[CH_3{}^{18}O]^+$	33.0221
	$[C_2H_5O]^+$	45.0334
	$[C_2H_5{}^{18}O]^+$	47.0378
$H_2{}^{18}O+O_2$	$[C_2H_3]^+$	27.0229
	$[CH_3O]^+$	31.0179
	$[C_2H_5O]^+$	45.0336

Supplementary Table 9 | GC-MS results of CH_3CHO for various isotopically labeled reactants.

isotope	CH_3CHO	m/z
H_2O+O_2	$[CHO]^+$	29.0022
	$[C_2H_2O]^+$	42.0101
	$[C_2H_3O]^+$	43.0179
	$[C_2H_4O]^+$	44.0258
D_2O+O_2	$[CHO]^+$	29.0022
	$[C_2H_3O]^+$	43.0177
	$[C_2H_4O]^+$	44.0249
	$[C_2H_3DO]^+$	45.0314
	$[C_2H_2D_2O]^+$	46.0378
	$[C_2HD_3O]^+$	47.0446
	$[C_2D_4O]^+$	48.0484
$H_2O+{}^{18}O_2$	$[CHO]^+$	29.0021
	$[C_2H_2O]^+$	43.0100
	$[C_2H_3O]^+$	43.0179
	$[C_2H_4O]^+$	44.0257
	$[C_2H_5{}^{18}O]^+$	46.0299
$H_2{}^{18}O+O_2$	$[C_2H_2]^+$	26.0151
	$[CH^{18}O]^+$	31.0064
	$[C_2H_3O]^+$	43.0178
	$[C_2H_4O]^+$	44.0258
	$[C_2H_3{}^{18}O]^+$	45.0222
	$[C_2H_4{}^{18}O]^+$	46.0300

Supplementary Table 10 | GC-MS results of CH_3COOH for various isotopically labeled reactants.

isotope	CH_3COOH	m/z
H_2O+O_2	$[CHO]^+$	29.0023
	$[C_2H_3O]^+$	43.0179
	$[CHO_2]^+$	44.9972
	$[C_2H_4O_2]^+$	60.0207
D_2O+O_2	$[CHO]^+$	29.0023
	$[C_2H_3O]^+$	43.0178
	$[C_2H_2DO]^+$	44.0241
	$[CHO_2]^+$	44.9971
	$[CDO_2]^+$	46.0034
	$[C_2H_4O_2]^+$	60.0205
	$[C_2H_3DO_2]^+$	61.0268
	$[C_2H_2D_2O_2]^+$	62.0330
	$[C_2HD_3O_2]^+$	63.0394
	$[C_2D_4O_2]^+$	64.0456
$H_2O+{}^{18}O_2$	$[C_2H_3O]^+$	43.0179
	$[CHO_2]^+$	44.9972
	$[C_2H_4O_2]^+$	60.0207
	$[C_2H_4O{}^{18}O]^+$	62.0249
$H_2{}^{18}O+O_2$	$[C_2H_3O]^+$	43.0179
	$[C_2H_3{}^{18}O]^+$	45.0221
	$[CHO{}^{18}O]^+$	47.0014
	$[CH{}^{18}O_2]^+$	49.0056
	$[C_2H_4O_2]^+$	60.0207
	$[C_2H_4O{}^{18}O]^+$	62.0248
	$[C_2H_4{}^{18}O_2]^+$	64.0291

Supplementary Table 11 | Calculated Reaction energetics of each elementary step for Rh_1/AC-SNI on the reaction path of producing ethanol with O_2 participating in the reaction.

Reaction coordination	state	ΔG (eV)	ΔE (eV)
A1	$O_2 + CH_3CH_3$	0	-
TS1	$2O^* + CH_3CH_3$	1.35	1.35
A2	$2O^* + CH_3CH_3^*$	1.11	-0.24
TS2	TS1	1.96	0.85
A3	$O^* + OH^* + CH_2CH_3^*$	0.99	-0.97
TS3	TS2	1.20	0.21
A4	$O^*+CH_3CH_2OH^*$	-1	-2.2
A5	$O^*+ CH_3CH_2OH^*$	-0.63	0.37
A6	CH_3CH_2OH	-1.16	-0.53

Supplementary Table 12 | Calculated Reaction energetics of each elementary step for Rh_1/AC-SNI on the reaction path of producing ethanol with H_2O participating in the reaction.

Reaction coordination	state	ΔG (eV)	ΔE (eV)
B1	H_2O +CH_3CH_3	0	-
TS1	TS1	0.44	0.44
B2	OH*+CH_3CH_3	0.26	-0.18
TS2	TS2	3.59	3.33
B3	H_2O*+ CH_3CH_2*	0	-3.59
B4	HO*+ CH_3CH_2*	0.17	0.17
TS3	TS3	0.88	0.71
B5	CH_3CH_2OH*	-1.16	-2.04
B6	CH_3CH_2OH	-1.2	-0.04

Supplementary Table 13 | Calculated Reaction energetics of each elementary step for Rh_1/AC-SNI on the reaction path of producing acetaldehyde with H_2O participating in the reaction.

**Reaction coordination**	**state**	**ΔG (eV)**	**ΔE (eV)**
C1	H_2O +CH_3CH_3	0	-
TS1	H_2O* +CH_3CH_3	0.44	0.44
C2	OH*+CH_3CH_3	0.36	-0.08
C3	O*+CH_3CH_3	0.73	0.37
C4	O*+CH_3CH_3*	0.61	-0.12
TS2	TS2	1.35	0.74
C5	OH*+CH_3CH_2*	0.12	-1.23
C6	OH*+CH_2CH_2*	-1.84	-1.96
TS3	TS3	-1.55	0.29
C7	CH_2CH_2OH*	-1.41	0.14
C8	CH_3CHO*	-2.56	-1.15
C9	CH_3CHO	-2.87	-0.31

Supplementary Table 14 | Calculated Reaction energetics of each elementary step for Rh_1/AC-SNI on the reaction path of producing acetaldehyde with O_2 participating in the reaction.

Reaction coordination	state	ΔG (eV)	ΔE (eV)
D1	O_2 +CH_3CH_3	0	-
TS1	TS1	1.35	1.35
D2	2O*+CH_3CH_3*	1.41	0.06
TS2	TS2	1.63	0.22
D3	O*+ OH*+CH_3CH_2*	0.99	-0.64
D4	2OH*+CH_2CH_2*	-0.88	-1.87
TS3	TS3	-0.23	0.65
D5	OH*+CH_2CH_2OH*	-0.47	-0.24
D6	OH*+CH_3CHO*	-1.65	-1.18
D7	CH_3CHO	-2.87	-1.22

Supplementary Table 15 | Calculated Reaction energetics of each elementary step for Rh_1/AC-SI on the reaction path of producing ethanol with O_2 participating in the reaction.

Reaction coordination	state	ΔG (eV)	ΔE (eV)
E1	O_2 +CH_3CH_3	0	-
TS1	TS1	2.03	2.03
E2	2O*+CH_3CH_3*	2.00	0.37
TS2	TS2	3.93	1.53
E3	O*+ OH*+CH_3CH_2*	2.05	-1.88
TS3	TS3	2.1	0.05
E4	O*+CH_3CH_2OH*	0.25	-1.85
E5	CH_3CH_2OH	-1.64	-1.89

Supplementary Table 16 | Calculated Reaction energetics of each elementary step for Rh_1/AC-NI on the reaction path of producing ethanol with O_2 participating in the reaction.

Reaction coordination	state	ΔG (eV)	ΔE (eV)
F1	O_2 +CH_3CH_3	0	-
TS1	TS1	1.6	1.6
F2	2O*+CH_3CH_3*	1.96	0.36
TS2	TS2	2.92	0.96
F3	O*+ OH*+CH_2CH_3*	2.01	-0.91
TS3	TS3	1.25	-0.76
F4	O*+CH_3CH_2OH*	0.07	-1.18
F5	CH_3CH_2OH	-1.33	-1.40
